# Medieval DNA from Soqotra points to Eurasian origins of an isolated population at the crossroads of Africa and Arabia

**DOI:** 10.1038/s41559-024-02322-x

**Published:** 2024-02-08

**Authors:** Kendra Sirak, Julian Jansen Van Rensburg, Esther Brielle, Bowen Chen, Iosif Lazaridis, Harald Ringbauer, Matthew Mah, Swapan Mallick, Adam Micco, Nadin Rohland, Kimberly Callan, Elizabeth Curtis, Aisling Kearns, Ann Marie Lawson, J. Noah Workman, Fatma Zalzala, Ahmed Saeed Ahmed Al-Orqbi, Esmail Mohammed Ahmed Salem, Ali Mohammed Salem Hasan, Daniel Charles Britton, David Reich

**Affiliations:** 1 Department of Human Evolutionary Biology, Harvard University, Cambridge, MA, USA.; 2 Department of Genetics, Harvard Medical School, Boston, MA 02115, USA.; 3 Chronicle Heritage, Phoenix, AZ 85004.; 4 Max Planck Institute for Evolutionary Anthropology, 04103 Leipzig.; 5 Howard Hughes Medical Institute, Harvard Medical School, Boston, MA, 02115, USA.; 6 Broad Institute of Harvard and MIT, Cambridge, MA, 02142, USA.; 7 General Organisation for Antiquities and Museums (GOAM), Hadiboh, Soqotra Island, Republic of Yemen.; 8 Arab Regional Centre for World Heritage, Manama, Kingdom of Bahrain.; 9 Environmental Protection Agency on Soqotra (EPA), Hadiboh, Soqotra Island, Republic of Yemen; 10 Independent Researcher, UK.

## Abstract

Soqotra, an island situated at the mouth of the Gulf of Aden in the northwest Indian Ocean between African and Arabia, is home to ~60,000 people subsisting through fishing and semi-nomadic pastoralism who speak a Modern South Arabian language. Most of what is known about Soqotri history derives from writings of foreign travelers who provided little detail about local people, and the geographic origins and genetic affinities of early Soqotri people has not yet been investigated directly. We report genome-wide data from 39 individuals who lived between ~650–1750 CE at six locations across the island and document strong genetic connections between Soqotra and the similarly isolated Hadramawt region of coastal South Arabia that likely reflects a source for the peopling of Soqotra. Medieval Soqotri can be modeled as deriving ~86% of their ancestry from a population like that found in the Hadramawt today, with the remaining ~14% best proxied by an Iranian-related source with up to 2% ancestry from the Indian sub-continent, possibly reflecting genetic exchanges that occurred along with archaeologically documented trade from these regions. In contrast to all other genotyped populations of the Arabian Peninsula, genome-level analysis of the medieval Soqotri is consistent with no sub-Saharan African admixture dating to the Holocene. The deep ancestry of people from medieval Soqotra and the Hadramawt is also unique in deriving less from early Holocene Levantine farmers and more from groups like Late Pleistocene hunter-gatherers from the Levant (Natufians) than other mainland Arabians. This attests to migrations by early farmers having less impact in southernmost Arabia and Soqotra and provides compelling evidence that there has not been complete population replacement between the Pleistocene and Holocene throughout the Arabian Peninsula. Medieval Soqotra harbored a small population that exhibited qualitatively different marriage practices from modern Soqotri, with first-cousin unions occurring significantly less frequently than today.

## INTRODUCTION

The four islands and two rocky outcrops of the Soqotra archipelago are situated in the northwest Indian Ocean at the mouth of the Gulf of Aden, approximately 380 kilometers south of the Arabian Peninsula and 250 kilometers northwest of the Horn of Africa. Soqotra, the largest island, is renowned for endemic biodiversity and rich resources which have long attracted mariners and traders. Today, it is home to approximately 60,000 people with distinctive language, culture, and traditions who subsist primarily through fishing, semi-nomadic pastoralism, and the cultivation of date palms. Modern Soqotri have cultural and genetic links to Arabia, southwest Asia, and east and north Africa, but most of what is known about their deeper history derives from the writings of foreign traders. Importantly, our understanding of Soqotra’s past is now also increasing through archaeological research (Jansen van Rensburg et al., 2022).

The Hadramawt, a region of southern Arabia consisting of present-day eastern Yemen and a small part of southern Saudi Arabia (and in pre-modern times also including part of western Oman), has the strongest evidence of close connections to Soqotra since at least the first centuries CE. Ceramics from Hajrya (located on Soqotra’s north coast) are similar to those from Wadi Hadramaut (a valley east of Aden in Yemen), while inscriptions in Hoq cave on Soqotra’s north coast attest to a strong southern Arabian influence beginning in the 2^nd^ century BCE (Strauch, 2012) and similarities between dolmen-shaped structures on Soqotra and those in the Hadramawt may suggest links as far back as the second half of the first millennium BCE (Naumkin, 1993; Weeks et al., 2002). The earliest account of people on Soqotra is in the *Periplus Maris Erythraei*, written in the mid-1^st^ century CE by an anonymous Greek trader from Egypt, who reported that the island (“*Dioscorida*”) was ruled by the Hadramawt Kingdom and leased to Arabian merchants (Casson, 1989), consistent with the hypothesis that the early inhabitants of Soqotra were South Arabian tribespeople who harvested frankincense during the height of the Hadramawt Kingdom’s power (Doe, 1992; Naumkin, 1993). Furthermore, the Soqotri language is closest to the Śḥerɛ̄t (‘Shahri’, ‘Jibbali’) language of Dhofar, the region of southern Oman bordering Al Mahrah; both are Modern South Arabian languages not closely related to the Arabic spoken elsewhere in Arabia (Morris et al., 2019).

Soqotra also played an important role in ancient long-distance trade networks and there is extensive archaeological evidence of foreign traders on Soqotra who visited the island while traveling along maritime routes that linked Arabia with Persia, India, and East Africa. The *Periplus* reported that Arab, Indian, and Greek traders settled on the north coast of Soqotra (Casson, 1989), while Cosmas Indicopleustes, a Greek traveler of the 6^th^ century CE, reported that Christian missionaries from Persia settled on the island in the mid-1^st^ millennium CE (Evers, 2014). Although texts from this period surely reflect the biases of their authors, they are worth noting especially when corroborated by other lines of evidence. Excavations of South Asian ceramics on the western end of Soqotra also attest to the presence of Indian traders at this time (Strauch, 2012). Inscriptions from Hoq cave record traders and travelers from Roman Egypt, Palmyra, Axum, Western India, Bactria, and Gandhara (an ancient civilization centered in northwest Pakistan) (Evers, 2014) between the 2^nd^ century BCE and 6^th^ century CE, drawn there by the supply of aloes, incense, and Dragon’s Blood (Indian cinnabar) (Strauch, 2012). The presence of foreign traders raises questions about the extent and nature of their interaction with local people, and ancient DNA provides a direct way to test if this included genetic exchange.

An Arabic version of the Abstract is available as Supplementary Note 1.

## RESULTS AND DISCUSSION

### Data overview

**Figure 1.**
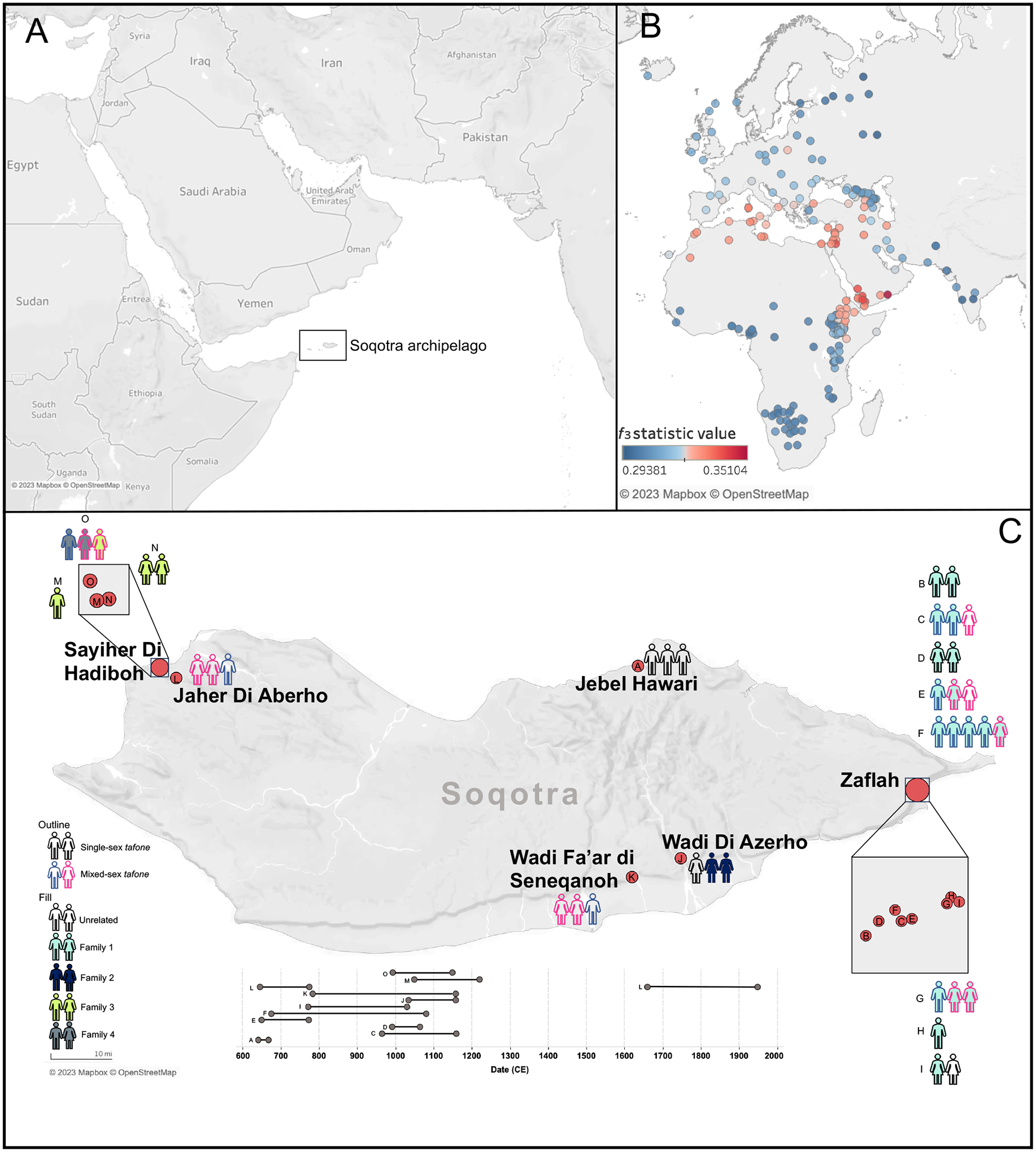
Overview of Soqotri burials with analysis of shared drift between medieval Soqotri and present-day groups. (A) Location of Soqotra. The Soqotra archipelago, situated at the crossroads of Arabia and Africa, is identified by the black box. (B) On the left, the 250 highest outgroup f_3_-statistics show shared drift between the medieval Soqotri and present-day populations genotyped on the Affymetrix Human Origins (HO) array using Karitiana as an outgroup. The color scale is in the bottom left corner, showing highest amount of allele sharing with the Yemeni_Desert group. Data are in Supplementary Data 8 and additional details about outgroup *f*_3_-statistic analysis are in Supplementary Note 7. (C) Burial patterns by sex and genetic relationships across 15 *tafoni* (A-O) on Soqotra. Legend is in the bottom left corner with additional information in Supplementary Data 14 and Supplementary Note 10. A timeline of *tafone* usage based on direct ^14^C dates and dates of lineal relatives with known relationships is included (bottom center).

We screened 50 skeletal elements from *tafoni* (sing. *tafone*) across six locations around Soqotra for DNA (Supplementary Notes 2 and 3). A *tafone* (“mesenaa’h” in Soqotri) is a geological formation resembling a small cave which has a round entrance and smooth, concave walls that forms part of the natural karst landscape. Both historically and today, Soqotri have used *tafoni* for storage, living quarters, animal shelters, and places for burying the dead (Bent, 1900; Doe, 1970, 1992; Jansen van Rensburg, 2018; Naumkin & Sedov, 1993; Naumkin, 1993; Shinnie, 1960; Weeks et al., 2002). We analyzed a tooth from each individual in most cases, and the DNA-rich petrous bone when possible (Pinhasi et al., 2015). Cortical bone from long bone shafts was analyzed if no cranial remains were present. Fragmented skeletal material was collected to minimize disruption to the burials. We obtained genome-wide ancient DNA data passing quality control for 39 individuals across 15 *tafoni* but excluded from most population genetic analysis seven individuals with very low coverage data (less than approximately 0.01 coverage of targeted positions) and an additional four individuals who are the lower-coverage first-degree relatives of another person in the dataset (Fig. 1A, Supplementary Data 1, 2). The individuals we report here lived as early as 641–668 calCE (1380±15 uncalibrated years before present, BP) and mostly during the Medieval period (7th-15th centuries CE), with one dating to the Modern Period (1660–1950 calCE (195±15 BP)) (Supplementary Data 3).

We merged the new ancient DNA data with previously published ancient data from Africans and Eurasians and present-day people from around the world whose genomes had been shotgun sequenced or enriched for sequences covering a set of ~1.2 million single nucleotide polymorphisms (SNPs). We also merged the new data with Affymetrix Human Origins (HO) SNP genotyping data from modern populations, including seven groups from Yemen (Lazaridis et al., 2014; Mallick et al., 2016; Vyas et al., 2017) and groups from other parts of Eurasia and Africa (Supplementary Note 4).

### The Soqotri gene pool changed little over a millennium

**Figure 2.**
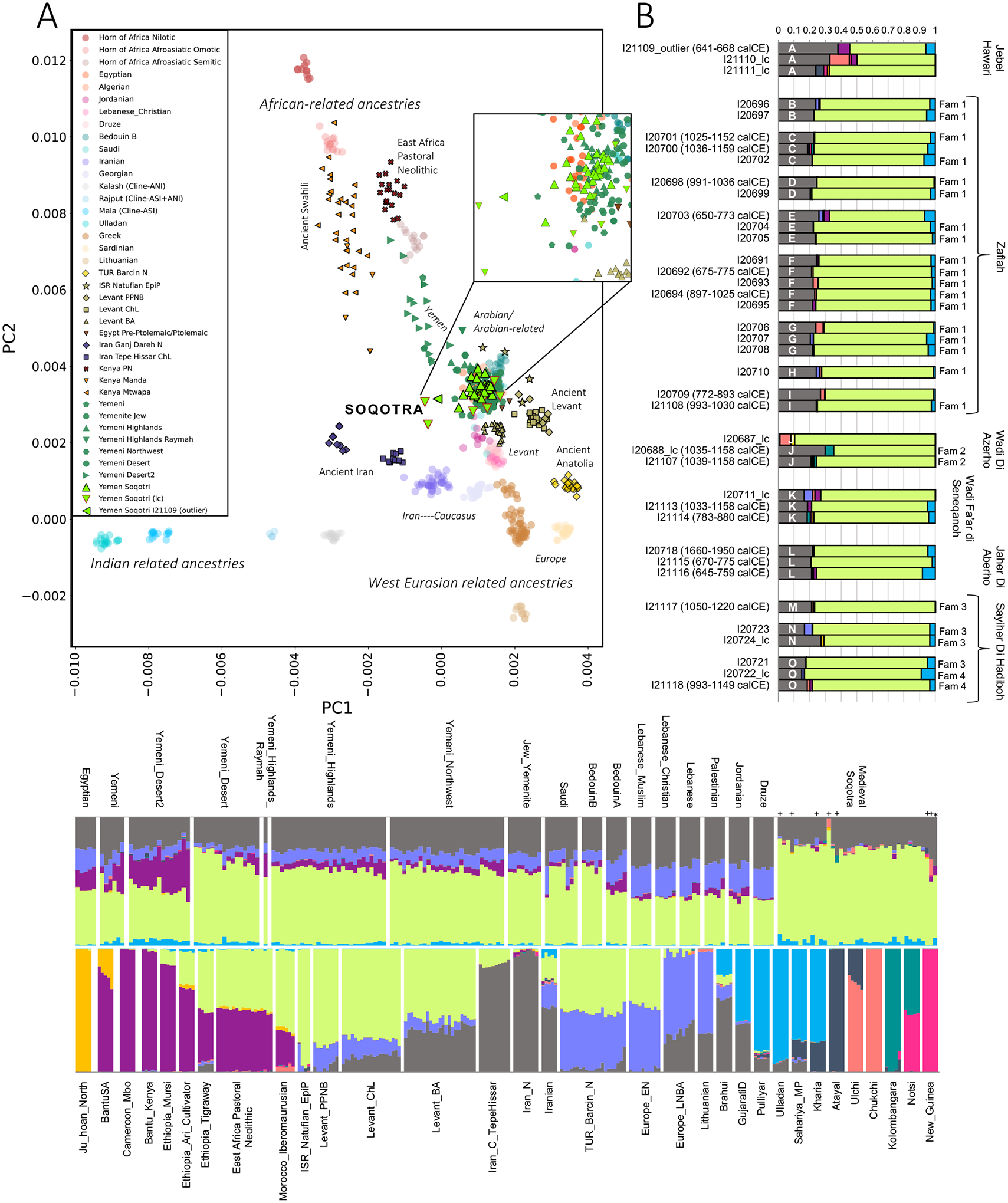
Qualitative analyses of medieval Soqotri DNA. (A) PCA. We computed axes with present-day individuals (Supplementary Note 4) and projected ancient individuals onto PCs 1 and 2. Inset provides a zoomed view of the Soqotri cluster. (B) Unsupervised ADMIXTURE at *K=*10 components for 39 Soqotri individuals. Individuals with <15,000 SNPs are marked “_lc” denoting low coverage; we include calibrated dates when available. Individuals are grouped by site starting on the northeast of the island and moving clockwise (site name written next to the plot), then by burial *tafone* (labeled A – O), see Supplementary Data 14). Members of four families are identified (Fam 1 – Fam 4, see Supplementary Data 13). (C) Additional results of unsupervised ADMIXTURE at *K=*10 components. Data for the Soqotri and relevant present-day groups from Egypt and the Near East are on top. The Soqotri are in the same order as in Panel B (top to bottom in Panel B corresponding to right to left in Panel C), with low coverage individuals denoted with a cross symbol and the outlier by an asterisk. A reference panel for the 10 components is on the bottom.

We used principal component analysis (PCA) to visualize how the genomes of the medieval Soqotri relate to previously published people (Fig. 2A; Supplementary Note 4). Most Soqotri fall in a tight cluster, suggesting that the presence of foreign traders across ~1,000 years had little genetic impact, or that any impact was relatively homogenous. Medieval Soqotri ancestry is qualitatively similar to that of some present-day Yemenite groups (Jew_Yemenite, Yemeni_Desert, Yemeni_Northwest, Yemeni_Highlands), but not others (Yemeni and Yemeni_Desert2) who appear to have a much higher proportion of sub-Saharan African-related ancestry. Relative to other ancient individuals, the medieval Soqotri fall nearest to ancient people from the Levant and ancient Egyptians with mostly Levantine-related ancestry but are shifted toward people with Iranian-related ancestry. Individual I21109 (dated to 641–668 calCE) is shifted furthest away from the main cluster. We use pairwise *qpWave* to evaluate whether every pair of individuals can be modeled as descending from the same ancestral sources relative to a reference population set (they form a clade relative to that set) (Supplementary Note 5) and find that except for I21109, all Soqotri individuals are consistent with forming a single genetic group (Extended Data Fig. 1; Supplementary Data 4). In pairwise comparisons, I21109 is rejected as forming a clade with all but five other individuals and is also rejected as forming a clade with a pool of the other Soqotri (p=0.003) so we removed this individual from the pool we call ‘Yemen_Soqotri’.

ADMIXTURE analysis shows that the medieval Soqotri, like most present-day groups from Yemen as well as Saudi and BedouinB, have a majority component (bright green) that is maximized in Late Pleistocene (Epipaleolithic) Natufian hunter-gatherers from the Levant (ISR_Natufian_EpiP in Fig. 2) and is inferred at high proportions in other Arabian and Levantine groups and some Africans from North and Northeast Africa who have substantial West Eurasian-related ancestry (Fig. 2B, 2C). The Soqotri additionally harbor an Iranian-associated component (grey; outlier I21109 has more of this component). Most distinctive about the Soqotri ADMIXTURE profile is their lack of an early European-like component (blue violet) otherwise found almost ubiquitously in people from Europe, Anatolia, the Levant, and Arabia; they also have less of a sub-Saharan African-related component (purple) than modern Arabians. Across the Soqotri, we detect a minor contribution from a component maximized in some South Asian populations, consistent with records of Indian traders on the island, although we cannot reject the hypothesis that this component is wholly Central Asian in origin (see “Medieval Soqotri have Central/South Asian-related ancestry”).

### The demographic impact of early Levantine farmers

To investigate the proportions of highly differentiated ancestries in the gene pools of Yemen_Soqotri and present-day people from the Arabian Peninsula (‘Arabians’) or Bedouins (‘Arabian-related’; Supplementary Note 4) we carried out ancestry modeling using *qpAdm*. We used populations deeply diverged in time as proxies for ancestry sources because few if any modern populations are unadmixed descendants of populations that lived even a millennium ago in this region (for example, most groups show evidence of recent African admixture (Hellenthal et al., 2014)).

Archaeological evidence has supported the hypothesis that Late Pleistocene hunter-gatherers associated with the Natufian culture inhabited the Arabian Peninsula as well as the Levant, and that early farmers carrying Neolithic Levantine/Anatolian-related ancestry later spread into Arabia and had some demographic impact, although the proportion of ancestry this population contributed is a topic of debate (Fedele, 2010; McCorriston & Martin, 2010; Uerpmann et al., 2010). Genetic analyses of present-day groups from the Levant and Arabia show that Natufian hunter-gatherers appear to share more alleles with Arabians than with Levantine populations; however, a model in which all Levantine/Anatolian-related ancestry in Arabians derives from a farmer-related source is also consistent with the data (Almarri et al., 2021).

To test the hypothesis that people in Arabia may harbor variable Levantine/Anatolian-related ancestry, we applied *qpAdm* in a framework that models present-day Arabian or Arabian-related groups and the Soqotri as mixtures of ancient populations shown in previous work to be useful surrogates for ancestry found in Eurasians (Lazaridis, Alpaslan-Roodenberg, et al., 2022a), plus an African-related source (Table 1). Fitting models required ancestry from ancient Levantine/Anatolian-, ancient Iranian-, and African-related sources. The Levantine/Anatolian ancestry in most Arabian or Arabian-related groups can be well-modeled using either a dual-source of Natufian hunter-gatherers (ISR_Natufian_EpiP) plus Neolithic Anatolian farmers (TUR_Barcin_N), or alternatively, using Neolithic Levantine farmers (Levant_PPNB) as a single source. This fit is consistent with Neolithic Levantine farmers representing a genetic intermediate between Natufians and Neolithic Anatolian farmers (Lazaridis, Alpaslan-Roodenberg, et al., 2022a, 2022b; Lazaridis et al., 2016); we fit a model for Neolithic Levantine farmers as having 60.5%±6.0% Natufian-related and 39.5%±6.0% Neolithic Anatolian farmer-related ancestry (p=0.30) (Supplementary Note 6).

In contrast to the majority of Arabian and Arabian-related groups, models using Neolithic Levantine farmers as a single source of the Levantine/Anatolian-related ancestry in Yemen_Soqotri are a poor fit (p<0.003). However, a four-source model where Natufians are a proxy for approximately two-thirds of the total Levantine/Anatolian-related ancestry and Neolithic Anatolian farmers are a proxy for the remaining one-third fits the data (p=0.13). In fact, a model without an African-related source fits the Soqotri data: in this more parsimonious model, Natufians fit as the source for over three-quarters of the total Levantine/Anatolian-related ancestry in the Soqotri gene pool (p=0.16). The inferred relative ancestry proportions of Natufian-related ancestry (~66% or ~78% in a four- and three-source model, respectively) and Neolithic Anatolian farmer-related ancestry for the medieval Soqotri are higher than the inferred proportions of Natufian-related ancestry (~54–59%) and Neolithic Anatolian farmer-related ancestry in the present-day Yemeni populations not from the Hadramawt. This implies that the poor fit is driven by either slightly more Natufian-related ancestry in Yemen_Soqotri, or alternatively, by excess genetic drift shared between Natufians and Yemen_Soqotri that is not shared to the same extent between Natufians and other Arabians. In either case, we detect the effects of substructure among Late Pleistocene and early Holocene Levantine populations, with medieval Soqotri receiving relatively more ancestry from ancient groups closer to the sampled Natufians.

Two present-day Arabian groups, Yemeni_Desert and Yemeni_Desert2, both from the Hadramawt region but split into sub-groups because of variation in sub-Saharan African-related ancestry proportions – also show a signal of excess Natufian-related ancestry (Table 1; Table S1 in Supplementary Note 6; Supplementary Data 5). Like Yemen_Soqotri, Yemeni_Desert cannot be fit with Neolithic Levantine farmers as a single Levantine/Anatolian-related source (p=0.0007) and instead can only be modeled with a dual source of ~42% Natufian-related ancestry and ~24% Neolithic Anatolian farmer-related ancestry (p=0.10). Results are qualitatively similar but less clear for Yemeni_Desert2, plausibly because of reduced power to discern distinctive ancestry due to a higher proportion of sub-Saharan African-related ancestry. The pattern of genetic similarity between these groups and the Soqotri is consistent with the archaeological evidence that Soqotra was likely peopled from, and maintained strong connections with, the Hadramawt (Naumkin, 1993), and is also consistent with studies of uniparental markers which point to similarities between the Soqotri and non-Arabic speaking groups living in the southern Arabian Peninsula (Černý et al., 2011). In contrast, groups from parts of Yemen that were plausibly in closer contact with other parts of the Arabian Peninsula and regions further afield, as well as a group of present-day Saudi, can be fit either with Neolithic Levantine farmers or with Natufians plus Neolithic Anatolian farmers, suggesting that much or all Levantine/Anatolian-related ancestry derives from a farmer-related source in these areas. This is consistent with a reduced displacement of hunter-gatherer-related ancestry in the ancestral gene pool of people who live in the Hadramawt and medieval Soqotra relative to people from other parts of Arabia, thus providing the strongest evidence to date that there has not been complete population replacement between the Pleistocene and Holocene throughout the Arabian Peninsula.

While the movements of Levantine farmers impacted the Soqotra archipelago relatively less than most of Arabia, the presence of both Anatolian Neolithic farmer- and Iranian-related ancestry in the Soqotri gene pool attests to additional gene flow. These ancestries could have arrived at least in part as a package from any of several different places. One possibility is that these ancestries reached Soqotra through later gene flow events from the Levant, as previous work has documented increased Anatolian Neolithic farmer- and Iranian-related ancestry in the Levant beginning in the Chalcolithic period, with a further increase in Iranian-related ancestry throughout the Bronze Age (Agranat-Tamir et al., 2020; Harney et al., 2018). We attempted to model Yemen_Soqotri using Chalcolithic or Bronze Age Levantine people as a source along with Natufians but found that additional Iranian-related ancestry was still required to fit the data (Supplementary Note 6, Supplementary Data 6). As Soqotra was also part of key maritime trade routes that connected Arabia with India and Persia to the east and the eastern Mediterranean to the west, it is possible that either or both parts of this trade network had some genetic impact on the medieval Soqotri gene pool. We attempted to model Yemen_Soqotri using Hellenistic/Roman-era Anatolians who have a mix of Anatolian/Iranian ancestry as a proxy for 1^st^ millennium CE Eastern Mediterranean traders as a source (Lazaridis, Alpaslan-Roodenberg, et al., 2022c), and also found that fitting models again required additional Iranian-related ancestry (Supplementary Note 6, Supplementary Data 6). While we cannot completely rule out that Levantine people or Eastern Mediterranean traders had some genetic impact on the medieval Soqotri, our results are consistent with the Soqotri receiving additional Zagros and/or Caucasus-related gene flow either directly from those regions or through an intermediate source not studied here.

### Medieval Soqotri have little sub-Saharan African ancestry

While there is archaeological evidence of African influence on Soqotra – for example, the Aksumite conquest is directly attested by inscriptional evidence in Hoq cave and imagery at Dahaisi cave (Jansen van Rensburg, 2018) – our data argue against substantial sub-Saharan African admixture into the medieval Soqotri gene pool. Specifically, ancestry models fit without an African-related source at all (African-related ancestry proportions overlap zero at two standard errors in a four-source model, while a three-source model excluding an African-related source also fits; Table 1).

We tested the robustness of a three-source ancestry model by rotating individuals or groups with sub-Saharan African-related ancestry into the reference set (Supplementary Data 7; Supplementary Note 6) and confirm that there is no genome-wide signal of African-related ancestry that cannot be completely represented by Natufians, a group which has affinity to ~15,000-year-old Iberomaurusian hunter-gatherers from Morocco (van de Loosdrecht et al., 2018).

With mitochondrial DNA (mtDNA) we find the only evidence for African ancestry in medieval Soqotra. Specifically, we identify one individual belonging to African-associated haplogroup L3h2, today distributed primarily within northern parts of East Africa and Soqotra (Soares et al., 2012); all other medieval Soqotri belong to the Eurasian-associated N and R branches of the mtDNA phylogeny (Supplementary Data 2), attesting to the strong Eurasian genetic connection that is also detected in mtDNA data from present-day people (Černý et al., 2016).

### Medieval Soqotri have Central/South Asian-related ancestry

To test which populations share the most genetic drift with Yemen_Soqotri, we computed outgroup *f*_3_-statistics using an Indigenous population from Brazil (Karitiana) as Outgroup rather than an African population (like Mbuti) to isolate the non-African part of the shared genetic drift and avoid the confounding effects of Holocene era African-related gene flow (Fig. S6 shows results with Mbuti as Outgroup). This analysis confirms that the medieval Soqotri share more drift with Yemeni_Desert than any other present-day genotyped population (Fig. 1B; Supplementary Note 7; Supplementary Data 8). D-statistics corroborate this strong relationship: *D*(Outgroup, Yemen_Soqotri; *X*, Yemeni_Desert) and *D*(Outgroup, Yemeni_Desert; Yemen_Soqotri, *X*) show that Yemen_Soqotri shares more alleles with Yemeni_Desert than with any other population (|Z|>8.5 and 7.2, respectively), while *D*(Outgroup, *X*; Yemen_Soqotri, Yemeni_Desert) passes the threshold for significance (max Z=4.3) only if a population with sub-Saharan African-related ancestry is *X* (Supplementary Data 9). We show with *qpAdm* that Yemeni_Desert has ~3% additional West African-related and ~18% Anatolian-related ancestry when Yemen_Soqotri is used as a proxy ancestry source (p=0.12) (Supplementary Note 6). Present-day Soqotri genomes are needed to determine if and to what extent these ancestries are found on Soqotra today.

Approximately 86% of medieval Soqotri ancestry can be modeled by Yemeni_Desert, with an additional ~14% proxied by a group with Iranian-related ancestry that has up to 2% contribution from the Indian sub-continent (modeled using a group with minimal Iranian-related ancestry like Pulliyar) (Supplementary Note 6). While this ancestry can be completely modeled with present-day Iranian people as a surrogate, an artificially-produced population mixed with 80% or more Iranian ancestry and the remainder Indian ancestry also provides working models (Supplementary Note 6). While our ability to discern specifically Indian-related ancestry is constrained by the Iranian-related ancestry shared between Central Asian and many South Asian populations, our data are consistent with a proportion of Indian-related ancestry in the Soqotri gene pool that is close to our limit of definitive detection. mtDNA haplogroup U2b2, which is nearly exclusive to South Asia (Narasimhan et al., 2019; Shinde et al., 2019), further suggests a Soqotri matrilineal connection to this region. Possible Iranian matrilineal ancestry is documented by the presence of haplogroup R2c in five ancient Soqotri individuals (Derenko et al., 2013). The high frequency of R2c in medieval Soqotra is distinct from the present-day mtDNA landscape, where most R haplotypes are part of the R0a clade hypothesized to have arisen in Arabia (Al-Abri et al., 2012; Černý et al., 2009; Gandini et al., 2016).

### First cousin unions were infrequent in medieval Soqotra

Like many other ethnically Muslim groups, modern Soqotri have a cultural preference for marriage between cousins. At least ~35% and up to ~40% of married respondents in an ethnographic study of Soqotri today were part of a first cousin union (this range reflects ~5% of marriages classified as occurring with a “father’s/mother’s relative” in Naumkin, 1993), with a preference for patrilineal orthocousin marriage (Naumkin, 1993). To determine if mate preference was similar in the past, we examined runs of homozygosity (ROH), segments of the genome where the genetic material inherited from both parents is identical, in 14 medieval Soqotri individuals of sufficient coverage (>0.3x coverage of targeted autosomal SNPs) (Supplementary Data 10; Supplementary Note 8) (Ringbauer et al., 2021). Long ROH segments (>20 centimorgans, cM) are indicative of parental relatedness within a few generations, while shorter ROH (4–8cM) indicate relatedness deeper in the pedigree, reflecting a limited mating pool (Ceballos et al., 2018; Ringbauer et al., 2021).

Over half of the medieval Soqotri have at least one long ROH, which have an appreciable chance of arising when parents are related at the level of fourth cousins or closer, indicating that many unions among medieval Soqotri were among people who belonged to the same extended family (see Figure S16 of Ringbauer et al., 2021). ROH data also provide evidence for an absence of close kin unions, defined here as first cousins or closer. I20705 from Zaflah, who has the largest amount of ROH_>20cM_ in our dataset (80.3cM ROH_>20cM_, with a max length of 34.3cM) is at the lower end of the range expected for the offspring of first cousins (26^th^ percentile from the perspective of summed ROH_>20cM_; 29^th^ percentile from the perspective of the longest segment; Supplementary Data 15). That the individual with the most ROH_>20cM_ is at the low end of the range expected for first cousins is surprising under the null hypothesis that the rate of first cousin unions in medieval times is the same as today. Specifically, in a sample of 14 individuals, we would expect about five to be the offspring of first cousin unions using the modern rate and would expect that the individual with the most ROH would be at a higher percentile of the first cousin distribution.

**Figure 3.**
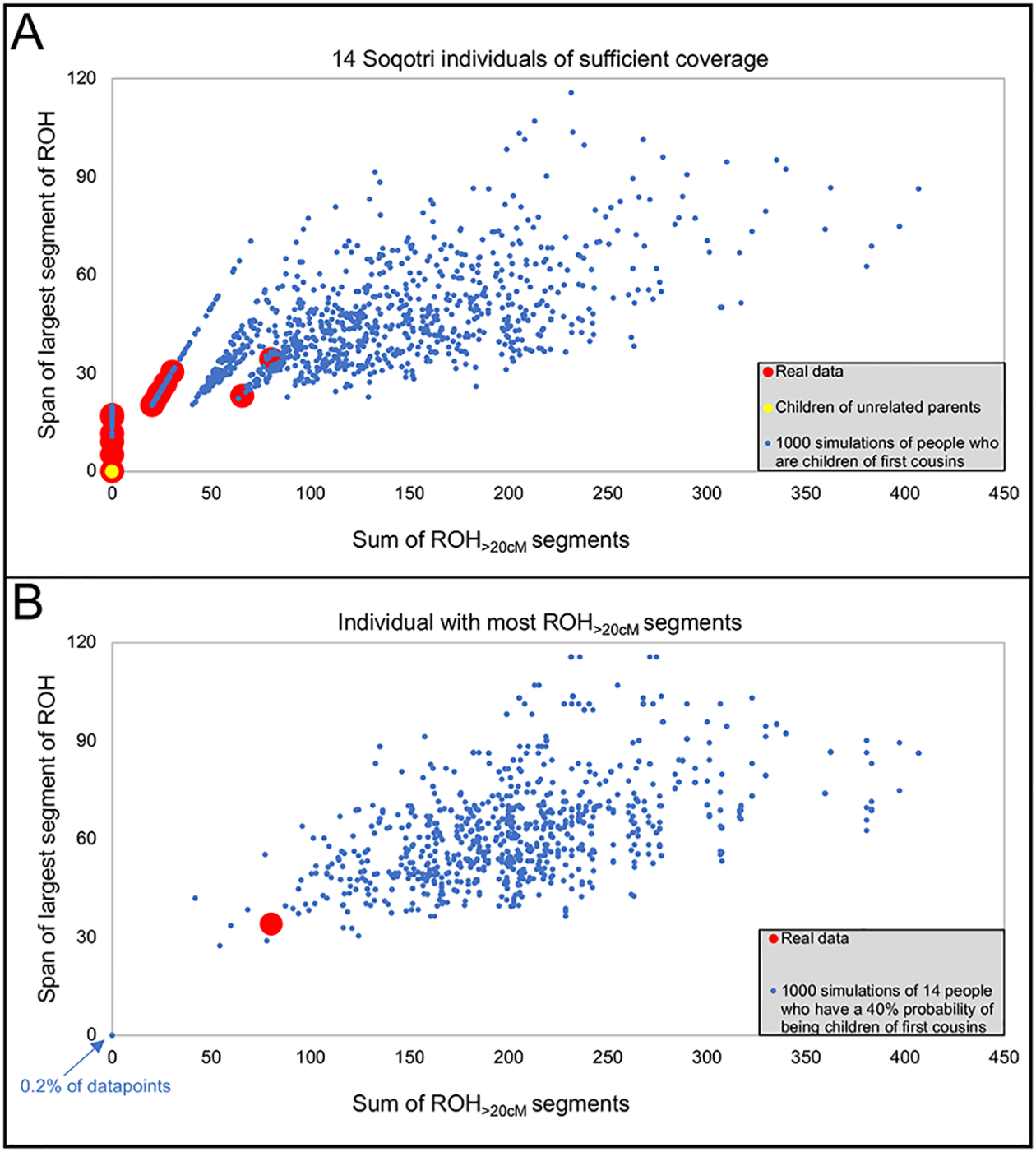
Medieval Soqotri had fewer long ROH than expected from modern ethnographic studies. We focused on long ROH (>20cM) as it is unlikely to arise except for unions of people in the same extended family, and hence inferences based on it are relatively unaffected by artifacts due to small island population sizes. (A) 1000 simulations of individual ROH profiles. We compare the real data to 1000 simulations of people whose parents are first cousins, under the simplifying assumption that all other genealogical relationships between peoples’ parents are very deep in the past. Treated as a single hypothesis test, I20705 (who has the most ROH out of the 14 ancient individuals for which we had ROH results) is in the p=0.26 percentile (one-sided test) of simulations with respect to the total summed ROH>20cM, and the p=0.29 percentile (one-sided test) of simulations with respect to the largest ROH segment. While this is in the range expected for the product of a first cousin union when treated as a single hypothesis test, it is surprisingly low given that modern ethnographic rates would predict about five first cousin unions in the data. (B) 1000 simulated sets of 14 individuals. We compare the maximum ROH observed in 1000 simulations of 14 individuals assuming the modern ethnographic rate of 40% first cousin unions and find that the real data is at the p=0.007 percentile (one-sided test) of simulations with respect to the total summed ROH>20cM, and p=0.009 percentile (one-sided test) of simulations with respect to largest ROH segment.

We carried out simulations to assess the proportion of first cousin unions in a sample of 14 individuals where all individuals have less ROH_>20cM_ than the maximum in our data (Fig. 3). Simulations show that the data are incompatible with the modern value of 40% cousin unions (p=0.007), and instead the p=0.05 threshold corresponds to a maximum of 26% first cousin unions (Supplementary Note 8). The data are also compatible with no first cousin unions among the 14 medieval Soqotri: the total ROH_>20cM_ in I20705 is in the 95^th^ percentile expected for second cousin unions (81^st^ percentile from the perspective of the largest single segment), which is unsurprising in a scenario which assumes that our sample of 14 medieval individuals includes multiple second cousin unions (for caveats, see Supplementary Note 8).

Consistent with a founder effect and consanguineous unions, there is low diversity in Y chromosome haplogroups among medieval Soqotri males: all except one belong to J2-M172 and specifically to the same branch of J2b-Z534 (outlier I21109 belongs to J1a2a1a2d2b-Z2324). The near-ubiquity of J2 haplogroups and paucity of J1 lineages is distinct from most of present-day mainland Yemen, where J1-M267 haplogroups dominate (Cadenas et al., 2008). This is consistent with the differences in the population history between Soqotra and most of the Arabian Peninsula that we identify through deep ancestry modeling (Supplementary Note 9).

We used the distribution of ROH among medieval Soqotri individuals with sufficient coverage to estimate an effective community size (*N*_e_) of 668 individuals (95% confidence interval [CI] 550–828 individuals). An estimate of *N*_e_ only in the hundreds of individuals provides further evidence of the relative isolation of the Soqotri population: despite a location along a key trade route, mating took place in a limited group resulting in a signal of genomic insularity, such as has been observed in other island communities (e.g., Ariano et al., 2022) (estimates for some present-day groups in Supplementary Data 11).

### No evidence of sex segregation in burial *tafone* on Soqotra

A long-standing subject of debate in the archaeology of Soqotra is whether *tafone* that had multiple interments were segregated by sex (Bent, 1900; Doe, 1970, 1992; Naumkin, 1993; Shinnie, 1960; Weeks et al., 2002; Wellsted, 1840). While collective burials in caves have been recorded in Saudi Arabia as well as in mainland Yemen, little is known about whether they represent families or tribe members (Luciani et al. 2018).

Ancient DNA provides an opportunity to resolve these questions. We determined genetic sex for the 39 Soqotri individuals in our dataset, 21 who were consistent with being genetically female (XX) and 18 who were consistent with being genetically male (XY) (Supplementary Data 2). Leveraging a method that compares the mean mismatch rate of all autosomal SNPs covered for both individuals (Olalde et al., 2019) as well as identity-by-descent (IBD)-based methods (Supplementary Data 12), we identify four genetic families (Fig. 2C) where people share relationships as close as first-degree and as distant as fifth- to seventh-degree (Supplementary Data 13). Seven out of 13 *tafoni* with the remains of multiple individuals contained members of both sexes, providing no support for a preference of placing individuals of the same sex together in death (Supplementary Data 14; Supplementary Note 10). Closely related individuals were sometimes, but not always, interred in the same *tafone*. The absence of relatives buried across sites is consistent with a preference for returning people to their home burial area for interment if they died elsewhere.

## Discussion

Genome-level data from medieval Soqotri people shines new light on an isolated population with very little sub-Saharan African-related ancestry and strong genetic links to the Hadramawt region. The medieval Soqotri had relatively more ancestry from ancient groups closer to Late Pleistocene Natufian huntergatherers, a signal also documented in present-day groups in the Hadramawt; in contrast, other present-day Arabian groups can be modeled as deriving all of their Levantine/Anatolian-related ancestry from a Neolithic farmer-related source. The reduced displacement of Natufian-related ancestry in Soqotra and the Hadramawt provides strong evidence that there has not been complete population replacement between the Pleistocene and Holocene throughout the entirety of the Arabian Peninsula.

The Soqotri gene pool remained stable over a millennium despite extensive archaeological evidence of foreign presence on the island, consistent with Soqotri oral history referring to the plateaus of the island interior that served as a refuge for local people to escape the attentions of foreign traders. Although traders and mariners were drawn to Soqotra by its rich resources and location along major Indian Ocean trade routes, they could arrive only by sea and rarely moved from coastal areas. Despite the remarkable archaeological evidence of foreign presence such as the Indian *Brāhmī*, Ethiopian Ge’ez, Roman Egyptian, and Palmyrene inscriptions within Hoq cave dating between the 2^nd^ century BCE and the 6^th^ century CE, the Soqotri gene pool can be ~86% modeled with Yemeni_Desert as a proxy source, suggesting migration from the Hadramawt had a dominant influence shaping the medieval Soqotri gene pool. How the remaining ancestry that is best proxied by an Iranian-related source with up to 2% ancestry from the Indian subcontinent was introduced to the Soqotri gene pool is unclear but is plausibly linked to the extensive maritime trade network that connected Arabia with Persia and India as well as with the eastern Mediterranean. Although the Indian-related ancestry in the Soqotri gene pool is close to or below our limit of definitive detection, archaeological and historical evidence support extensive Indian activity on Soqotra. For example, Indian inscriptions in Hoq cave are nearly all written in informal varieties of Brāhmī script and in Sanskrit or vernacularized Sanskrit dated to the 2^nd^-4^th^ centuries CE (Strauch, 2012), while text from the *Periplus* records India’s provision of female slaves to Soqotra (Dridi, 2002; Dridi & Gorea, 2003; Robin & Gorea, 2002). Both the *Periplus* and epigraphic evidence specifically provide a record of traders coming from Barygaza (present-day Broach) and Hāthab in Gujarat on the northwest coast of India; epigraphic evidence also suggests Indians traveling to Soqotra from the Malabar coast of southwest India (Strauch, 2012; Strauch & Bukharin, 2004), while a single text is written in the Kharosthī script exclusively used in the northwest of India (Strauch, 2016). The presence of Indians on Soqotra is continuously mentioned from the 10^th^-13^th^ centuries, although they were increasingly portrayed as pirates who at least had some children with the coastal population (Al-Mujāwir & ibn Yaʿqūb, 2008).

We also find evidence of a shifting practice toward an increased frequency of cousin marriage from medieval to present-day Soqotra. This reduction in the rate of first cousin unions would be consistent with a similar pattern observed in medieval northwest Pakistan where none of four individuals buried in a Muslim context from 900–500 BP had evidence of close parental relatedness, in sharp contrast to modern patterns of high rates (Ringbauer et al., 2021). Considered together, these results not only suggest a relatively recent shift in cultural practice in Soqotra toward a preference for first-cousin marriages, but also raise the possibility that this has been part of a broader cultural change toward preferences for such marriages in the Muslim world. In particular, it is possible that the spread of the practice of “Bint’amm” marriages between paternal cousins—which is common in the Muslim world today and has been hypothesized to have spread from the Arabian Peninsula with the movement of Arab tribes in the 7^th^-8^th^ centuries (Bittles & Hamamy, 2010; Korotayev, 2000; Stern, 1939) became so widespread only during the medieval-to-modern transition. These results are tentative and an important topic for future work is to test this hypothesis in other parts of the Islamic world where first cousin unions are common today.

Finally, a long-standing subject of debate in the archaeology of Soqotra regards burial patterns of people in natural *tafone* on Soqotra, first documented in the 17^th^ century by the Carmelite P. Vincenzo who mentioned that each family on Soqotra had a cave in which they placed their deceased (Yule & Cordier, 1993). This practice of burying relatives in caves was reported to reflect family members having no strength left to dig graves due to epidemics or drought. However, until now, it has been unclear whether *tafone* were segregated by sex. We show that seven out of 13 *tafoni* containing the remains of multiple individuals included individuals of both sexes, and provide evidence of familial as well as matrilineal and patrilineal relationships among people buried in a *tafone* which is consistent with a strong preference for interment in one’s local burial area in a family *tafone* if possible, or with other tribespeople in other cases. An important opportunity for obtaining further insights will come from collecting genome-wide data from modern Soqotri people, which would make it possible to understand how they relate to people who lived on the island a millennium ago.

## METHODS

### Community Engagement.

Ancient DNA can be used to investigate geographic origins, genetic affinities, and population structure of people who lived long ago. In Soqotra, an important part of our project was to directly involve the local community in telling the story of the people who lived in the same region over the last two millennia. Long before ancient DNA analysis began, we established a relationship with multiple local people, specifically the Soqotra Governorate, the Director of the General Organization of Antiquities and Museums (GOAM) Soqotra, local heritage practitioners from the Soqotra Heritage Project (SHP), and villagers living in the areas from which samples were taken. Collection of human skeletal material for this study was undertaken by a team of women and men from the SHP and GOAM who received education and training in the identification, recording, and assessment of the burials from which samples were taken. This included information about how and why samples of skeletal material are collected for ancient DNA studies. This knowledge, coupled with direct involvement in recording and collecting samples, provided local people with a deeper understanding of the fieldwork and helped them better communicate and educate local governmental officials, communities, students, and school children during the various outreach activities that were carried out during and after fieldwork was completed. Permissions for the collection of samples were provided by GOAM and village leaders (*sheiks*), who either accompanied the team or provided guides to help in the location and identification of areas where sampling was permitted. Permits for export and analysis of all skeletal samples were obtained from GOAM (Supplementary Note 2).

### Laboratory work and bioinformatics analysis.

In ancient DNA-dedicated clean room facilities at Harvard Medical School, we generated ~40mg of powder from bone and tooth samples, specifically targeting skeletal elements known to be especially DNA-rich (Hansen et al., 2017; Pinhasi et al., 2015; Pinhasi et al., 2019). Details about sample selection can be found in Supplementary Note 2, and the skeletal element used for each individual can be found in Supplementary Data 1 and 2. We performed DNA extraction using silica-coated magnetic beads to support robotic clean-ups (Rohland et al., 2018). From the extracts, we prepared dual-barcoded libraries using double-strand ligation, and we treated these libraries with uracil-DNA glycosylase (UDG) in a modified partial UDG preparation (‘partial’), leaving a reduced damage signal at both ends (5′ C-to-T, 3′ G-to-A) (Rohland et al., 2015, additional details in Supplementary Data 1). To generate SNP capture data, we used in-solution target hybridization to simultaneously enrich for sequences that overlap the mitochondrial genome and about 1.2 million genome-wide SNPs (Fu et al., 2015; Fu, Mittnik, et al., 2013; Haak et al., 2015; Mathieson et al., 2015). We then added two seven-base pair indexing barcodes to the adapters of each double-stranded library and carried out sequencing using an Illumina HiSeqX10 instrument with 2×101 cycles and reading the indices with 2×7 cycles.

We merged paired-end sequences, retaining reads that exhibited no more than one mismatch between the forward and reverse base if base quality was ≥20, or three mismatches if base quality was <20 prior to alignment. A custom toolkit (available at https://github.com/DReichLab/ADNA-Tools) was used for merging and trimming adapters and barcodes. Merged sequences were mapped to the Reconstructed Sapiens Reference Sequence (RSRS) (Behar et al., 2012) and the human reference genome version hg19 using the *samse* command in BWA 0.7.15-r1140 (Li & Durbin, 2009) with the parameters -*n 0.01, -o 2, and -l 16500*. Duplicate molecules were removed following alignment using the Picard MarkDuplicates tool of the Broad Institute (v.2.17.10; http://broadinstitute.github.io/picard/). We trimmed two terminal bases from the DNA fragments in all partial-UDG libraries to reduce damage-induced errors.

We evaluated the authenticity of the isolated DNA by retaining individuals with a minimum of 3% of cytosine-to-thymine substitutions at the end of the sequenced fragments (Rohland et al., 2015), point estimates of mtDNA contamination below 5% (estimates made with contamMix v.1.0–12; Fu, Meyer, et al., 2013), and point estimates of X chromosome contamination in males with sufficient coverage below 3% (estimates made with ANGSD v. 0.921–3-g40ac3d6 (htslib: 1.7) build(Jan 26 2018 13:16:35)) (Korneliussen et al., 2014) (Supplementary Data 1).

We determined SNPs by randomly sampling an overlapping read with minimum mapping quality of ≥10 and base quality of ≥20, generating ‘pseudo-haploid’ calls. Individuals with fewer than 20,000 covered SNPs out of ~1.24 million were excluded from quantitative analyses. One individual from each of four pairs of first-degree relatives in the dataset was excluded from population genetics analysis when the data were grouped; in all cases, we retained the higher coverage individual (Supplementary Data 1 and 2). Library information for all individuals studied as part of this work is in Supplementary Data 1, and we also report negative results (samples that failed to yield authentic ancient DNA) in this table to prevent duplicative efforts in the future.

### Radiocarbon dates and isotope data.

We obtained 18 accelerator mass spectrometry (AMS) radiocarbon dates (^14^C) from the Pennsylvania State University (PSU) Radiocarbon Laboratory. We sonicated all of the bone samples in successive washes of American Chemical Society (ACS) grade methanol, acetone, and dichloromethane for 30 minutes each at room temperature, followed by three washes in Nanopure water to rinse in order to remove possible contaminants (conservants and adhesives). We extracted bone collagen and purified using a modified Longin method with ultrafiltration (>30 kDa gelatin) (Kennett et al., 2017). If collagen yields were low and amino acids were poorly preserved, we used a modified XAD process (XAD Amino Acids) (Lohse et al., 2014). The method used for each sample can be found in Supplementary Data 3.

For quality assurance, we measured carbon and nitrogen concentrations and C/N ratios of all of the extracted and purified collagen/amino acid samples using a Costech elemental analyzer (ECS 4010). We evaluated sample quality by percentage of crude gelatin yield, percentage of C, percentage of N and C/N ratios before AMS ^14^C dating. C/N ratios for all directly radiocarbon samples except one (I20701) fell between 3.1 and 3.3, indicating excellent preservation (Van Klinken, 1999). We combusted collagen/amino acid samples (~2.1 mg) for 3 h at 900 °C in vacuum-sealed quartz tubes with CuO and Ag wires. Sample CO_2_ was reduced to graphite at 550 °C using H_2_ and a Fe catalyst, and we drew off reaction water with Mg(ClO_4_)_2_ (Santos et al., 2004). We pressed graphite samples into targets in aluminum boats and loaded them onto a target wheel and performed all ^14^C measurements using a modified National Electronics Corporation compact spectrometer with a 0.5 MV accelerator (NEC 1.5SDH-1). We corrected the ^14^C ages for mass-dependent fractionation with measured δ^13^C values (Stuiver & Polach, 1977) and compared with samples of Pleistocene whale bone (>48,000 ^14^C BP), late Holocene bison bone (~1,850 ^14^C BP), late AD 1800s cow bone and OX-2 oxalic acid standards. We calibrated ^14^C ages using OxCal v.4.4 (Bronk Ramsey & Lee, 2013) and the IntCal20 northern hemisphere curve (Reimer et al., 2020).

### Dataset assembly.

We merged the newly published ancient DNA data with previously published data from ancient Africans and Eurasians and present-day people from around the world whose genomes had been shotgun sequenced or enriched for sequences covering a canonical set of ~1,233,013 million SNPs (1240k data) (Fu et al., 2015; Fu, Mittnik, et al., 2013; Haak et al., 2015; Mathieson et al., 2015), as well as whole-genome sequence (WGS) data from ancient and present-day African and Near Eastern groups (Agranat-Tamir et al., 2020; Brielle et al., 2023; Fan et al., 2019; Fregel et al., 2018; Fu et al., 2014; Gallego-Llorente et al., 2015; Harney et al., 2018; Jones et al., 2015; Kılınç et al., 2016; Lazaridis, Alpaslan-Roodenberg, et al., 2022a, 2022b, 2022c; Lazaridis et al., 2016; Lipson et al., 2020; Lipson et al., 2022; Mallick et al., 2016; Meyer et al., 2012; Narasimhan et al., 2019; Prendergast et al., 2019; Prüfer et al., 2014; Raghavan et al., 2014; Schlebusch et al., 2017; Schuenemann et al., 2017; Sirak et al., 2021; Skoglund et al., 2015; Skoglund et al., 2017; van de Loosdrecht et al., 2018). We also merged the new data with Affymetrix Human Origins (HO) SNP genotyping data (597,573 SNPs) which allows us to leverage data from a more diverse range of present-day populations from Eurasia and Africa (Biagini et al., 2019; Brielle et al., 2023; Broushaki et al., 2016; Changmai et al., 2022; Flegontov et al., 2019; Jeong et al., 2019; Lazaridis et al., 2016; Lazaridis et al., 2014; Lipson et al., 2018; Lipson et al., 2020; Liu et al., 2020; López et al., 2021; Nakatsuka et al., 2017; Patterson et al., 2012; Pickrell et al., 2012; Qin & Stoneking, 2015; Skoglund et al., 2016; Skoglund et al., 2017; Vyas et al., 2017; Wang et al., 2021). Whenever we needed to co-analyze 1240k data and HO array data, we restricted to SNPs also covered on the HO SNP array.

### Uniparental haplogroups.

We called mitochondrial DNA (mtDNA) haplogroups using HaploGrep2 (Weissensteiner et al., 2016), reported in Supplementary Data 2. We determined Y chromosome haplogroups using both targeted SNPs as well as off-target sequences that aligned to the Y chromosome based on comparisons to the Y chromosome phylogenetic tree with Yfull version 8.09 (https://www.yfull.com/). We provide two notations for Y chromosome haplogroups in Supplementary Data 2. The first uses a label based on the terminal mutation, while the second describes the associated branch of the Y chromosome tree based on the nomenclature of the International Society of Genetic Genealogy (http://www.isogg.org) version 15.73 (July 2020).

### PCA.

We performed PCA with *smartpca* v.18200 (Patterson et al., 2006) and the parameters lsqproject: YES’, ‘newshrink: YES’, ‘numoutlieriter: 2’, and ‘hiprec: YES’. We computed eigenvectors of the covariance matrix of SNPs from 1198 present-day individuals from 63 modern populations genotyped on the Affymetrix Human Origins (HO) SNP array. We projected all other individuals (including ancient individuals) onto the first two principal components (PC1 and PC2). Full details of PCA setup are in Supplementary Note 4.

### ADMIXTURE (v.1.3.0).

We ran unsupervised ADMIXTURE (Alexander et al. 2009) using 2934 world-wide individuals either genotyped on the HO array or pulled down on the HO SNP set. We used *convertf* to transform the dataset from PACKEDANCESTRYMAP format to the PACKEDPED format for pruning in PLINK2 (Chang et al. 2015) and pruned SNPs based on linkage disequilibrium (LD), using the *indep-pairwise* option with a window size of 200 variants, a step size of 25 variants, and a pairwise threshold of 0.4. We ran three replicates of ADMIXTURE with random seeds with *K*=3 to *K*=12 ancestral reference populations and present *K*=10 based on low cross-validation errors with the goal of not overfitting.

### Genetic relatedness.

We tested genetic relatedness between every pair of newly reported individuals following the method described in (Olalde et al., 2019) and also applied IBD analysis on the X chromosome of males with sufficient coverage (>400,000 SNPs) to further specify the nature of genetic relationships (e.g., avuncular) and identify more distant relatives (e.g., 5^th^-7^th^-degree relatives). Results are in Supplementary Data 13, with discussion of genetic relationships in terms of burial groupings in 16 tafone in Supplementary Data 14. We use IBD analysis to show some additional more distant relationships amount eight individuals from Zaflah with sufficient coverage in Supplementary Data 12.

### Analysis of consanguineous genetic segments.

We identified runs of homozygosity (ROH) using the Python package hapROH (https://test.pypi.org/project/hapROH/) which uses 5008 genomes from the 1000 Genomes project haplotype panel (1000 Genomes Project Consortium, 2015) as the reference panel. We restricted our analyses to 14 medieval Soqotri individuals who had a minimum coverage of 300,000 SNPs and called ROH >4 centiMorgan (cM). We used the default parameters of hapROH which are optimized for ancient DNA data. In Supplementary Data 10 we report the total sum amount and number of ROH in length bins 4–8cM, 8–12cM, 12–20cM, and >20cM as well as the longest ROH block for each individual. We include comparative plots for Arabian and Arabian-related modern populations in Supplementary Note 8.

### *qpWave* (v.1540).

We used *qpWave* from ADMIXTOOLS (Patterson et al., 2012) to test cladality between every pair of medieval Soqotri individuals in our analysis dataset. We used ‘allsnps: YES’ which calls *qpfstats* to calculate the relevant f-statistics and set Han.SDG as the base population (‘basepop: Han.SDG’) for all *qpfstats* calculations (https://github.com/DReichLab/AdmixTools/blob/master/qpfs.pdf). We set ‘inbreed: NO’ but also added the ‘inbreedlistname’ parameter so that any population with more than one individual would instead be analyzed as if we set ‘inbreed: YES’. Results are in Supplementary Data 4, with additional details in Supplementary Note 5.

### *qpAdm* (v.1770).

We used the *qpAdm* framework (Haak et al., 2015) to model ancestry and estimate ancestry proportions. *qpAdm* uses *f*_*4*_-statistics to detect share drift between the population of interest (the ‘target’ population) and possible admixing source proxies relative to a set of differentially related outgroup populations (referred to as the ‘reference set’). It evaluates whether a population of interest can be plausibly modeled as descending from a common ancestor of one or more source populations and produces unbiased estimates of ancestry proportions from these sources (Haak et al., 2015). For models consistent with the data (p>0.05), qpAdm estimates proportions of admixture for the target population from the specified source populations without requiring an explicit model for how the reference populations are related. We used ‘allsnps: YES’ which calls *qpfstats* to compute f-statistics and set Han.SDG as the base population (‘basepop: Han.SDG’) for all *qpfstats* calculations (https://github.com/DReichLab/AdmixTools/blob/master/qpfs.pdf). We set ‘inbreed: NO’, but also added the ‘inbreedlistname’ parameter so any population with more than one individual would be analyzed as if we set ‘inbreed: YES’. Additional details are in Supplementary Note 6.

### f-statistics.

We computed *f*_*4*_-statistics using *qpDstat* (v.994) from ADMIXTOOLS (Patterson et al., 2012) with ‘f4 mode: YES’ and ‘inbreed: YES.’ We computed ‘outgroup’ *f*_*3*_-statistics using *qp3Pop* (v.670) from ADMIXTOOLS (Patterson et al., 2012) with ‘inbreed: YES.’ Additional details about *f*_*3*_-statistics as used in this work are in Supplementary Note 7.

## Extended Data

**Extended Data Figure 1.**
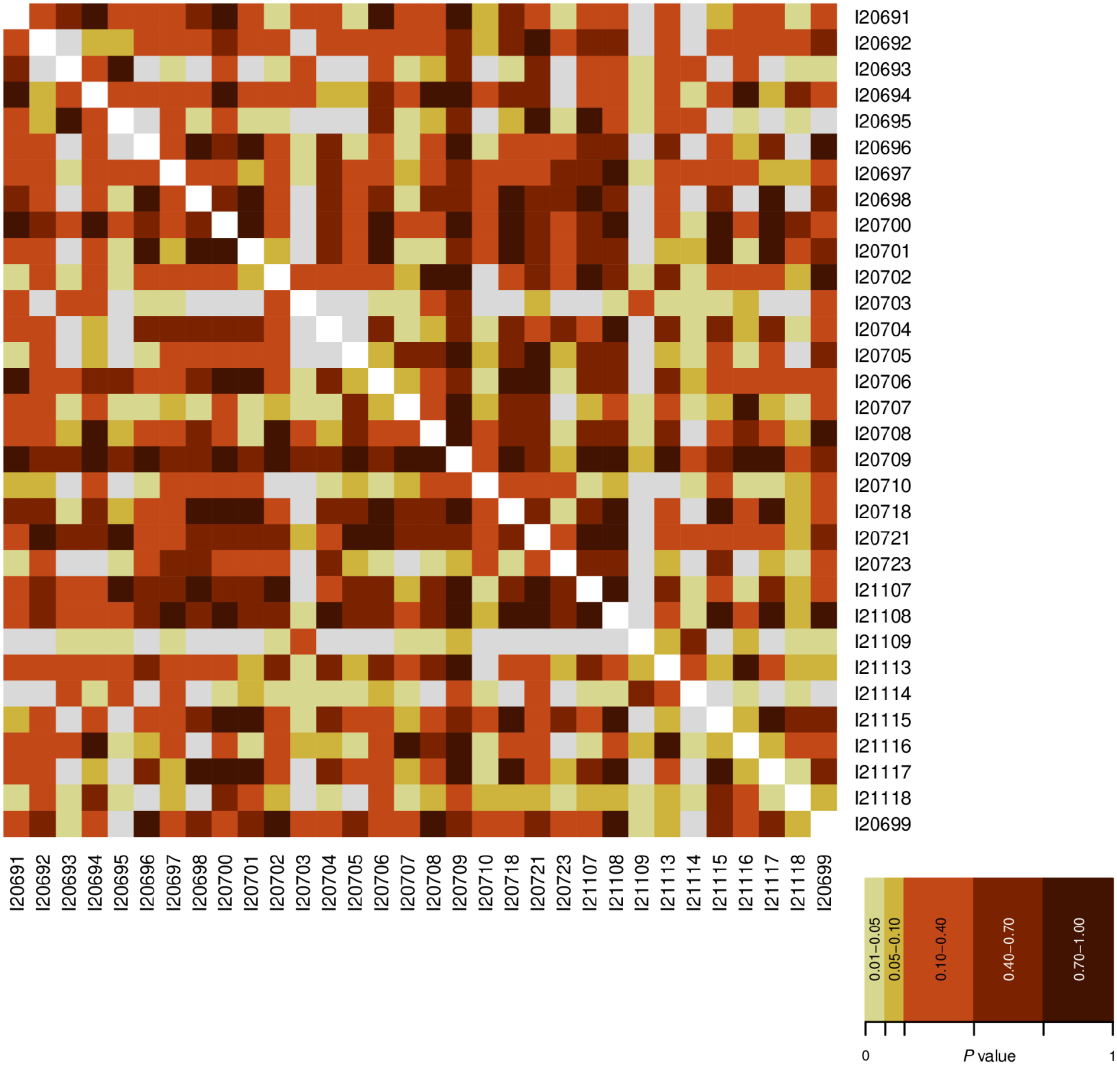
Heatmap of pairwise *qpWave* tests for 32 medieval Soqotri individuals with sufficient SNP coverage. Data are in Supplementary Data 4 and details are in Supplementary Note 5. We interpret pairwise model p-values >0.01 (colored cells) to be consistent with the two individuals forming a clade relative to the reference population set, and p-values <0.01 (grey cells) to be inconsistent.

## Supplementary Material

Supplementary Information

Supplementary Data

## Figures and Tables

**Table 1.** *qpAdm* models of the distal ancestry of present-day Arabian or Arabian-related groups and Yemen_Soqotri. We include ancestry proportions and one standard error. Results significant at p=0.01–0.05 are in italics, results significant at p>0.05 are in bold and italics (we interpret a p-value above 0.05 to be consistent with a plausible admixture model, while a p-value between 0.01 and 0.05 indicates a marginal fit of the model). The last column reports the percentage of Levantine/Anatolian-related ancestry contributed by a Natufian-related (ISR_Natufian_EpiP-related) source for each group.

	% ancestry (± % s.e.)				% ancestry (± % s.e.)					
Target	Levant_PPNB	Iran_N	Eth_4500 BP	p-value	ISR_Natuf_EpiP	Iran_N	TUR_Barcin_N	Eth_4500 BP	p-value	Proportion of Levant/Anatolian ancestry modeled with ISR_Natufian_EpiP
BedouinA	59.3 ± 2.4	33.1 ± 2.3	7.6 ± 0.5	*0.04174*	31.7 ± 5.5	35.5 ± 2.7	25.1 ± 4.9	7.8 ± 1.0	0.00740	55.81%
BedouinB	65.5 ± 2.6	30.4 ± 2.6	4.1 ± 0.6	***0.21748***	34.1 ± 5.6	32.7 ± 2.9	28.7 ± 5.1	4.5 ± 1.1	***0.10627***	54.30%
Saudi	61.9 ± 2.6	36.2 ± 2.6	2.0 ± 0.6	***0.36931***	32.0 ± 5.1	38.8 ± 2.9	26.9 ± 4.8	2.4 ± 1.0	***0.54233***	54.33%
Jew_Yemenite	63.2 ± 2.7	34.1 ± 2.7	2.7 ± 0.6	***0.21456***	33.6 ± 5.3	36.4 ± 2.9	27.0 ± 4.9	3.0 ± 1.0	***0.50612***	55.45%
Yemeni	48.8 ± 2.5	36.8 ± 2.5	14.4 ± 0.7	***0.14294***	26.4 ± 4.8	38.6 ± 2.7	20.5 ± 4.6	14.6 ± 1.0	***0.65806***	56.29%
Yemeni_Desert	67.5 ± 2.8	28.8 ± 2.8	3.7 ± 0.6	0.00072	41.9 ± 6.7	31.3 ± 3.5	23.4 ± 6.2	3.3 ± 1.2	***0.10329***	**64.17%**
Yemeni_Desert2	43.4 ± 2.4	28.5 ± 2.4	28.1 ± 0.6	0.00342	27.7 ± 5.9	31.4 ± 2.7	13.4 ± 5.2	27.5 ± 1.2	*0.02966*	**67.40%**
Yemeni_Highlands	62.9 ± 2.5	31.1 ± 2.5	6.1 ± 0.5	*0.01850*	35.4 ± 5.2	33.5 ± 2.8	25.1 ± 4.8	6.1 ± 1.0	***0.32483***	58.51%
Yemeni_Highlands_Raymah	61.4 ± 4.4	19.0 ± 4.4	19.6 ± 1.3	***0.27552***	34.2 ± 7.5	21.8 ± 4.5	24.4 ± 7.3	19.6 ± 1.7	***0.39685***	58.36%
Yemeni_Northwest	64.2 ± 2.5	31.9 ± 2.5	3.9 ± 0.6	*0.03131*	33.2 ± 4.9	33.9 ± 2.7	28.5 ± 4.5	4.3 ± 0.9	***0.45075***	53.81%
Yemen_Soqotri	65.6 ± 2.9	32.3 ± 2.8	2.1 ± 0.6	0.00264	42.0 ± 6.7	35.1 ± 3.5	21.3 ± 6.1	1.6 ± 1.2	***0.13450***	**66.35%**
Yemen_Soqotri (no African source)	67.6 ± 2.9	32.4 ± 2.9	-	0.00004	49.3 ± 5.2	36.6 ± 3.9	14.2 ± 5.1	-	***0.15674***	**77.64%**

## Data Availability

The aligned sequences are available through the European Nucleotide Archive under accession number PRJEB66485. Genotype data are available at https://reich.hms.harvard.edu/datasets. Any other relevant data are available from the corresponding authors upon reasonable request.
