# Supplementary material for: Medieval DNA from Soqotra points to Eurasian origins of an isolated population at the crossroads of Africa and Arabia
Source: Nat Ecol Evol. Author manuscript; Available in PMC 2024 Apr 12. (PMC11009077; doi:10.1038/s41559-024-02322-x)
Supplement: Supplementary Information [file NIHMS1961394-supplement-Supplementary_Information.pdf]

Supplementary Information

Medieval DNA from Soqotra points to Eurasian origins of an isolated population at the crossroads of Africa and Arabia

Supplementary Note 1: Arabic Abstract

Supplementary Note 2: Permissions and Sampling Protocols

Supplementary Note 3: Archaeological Site Summaries

Supplementary Note 4: PCA Setup

Supplementary Note 5: *qpWave*

Supplementary Note 6: *qpAdm* Modeling

Supplementary Note 67: Outgroup f_3-statistics

Supplementary Note 8: Analysis of Consanguineous Genetic Segments

Supplementary Note 9: Y Chromosome Haplogroups

Supplementary Note 10: Burial Groupings

Supplementary Note 1: Arabic Abstract

العنوان - Title

الحمض النووي من العصور الوسطى في سُقُطْرَى يشير إلى أصول أوراسية لسكان معزولين عند ملتقى أفريقيا والجزيرة العربية

المستخلص - Abstract

سُقُطْرَى، جزيرة تتميز بتنوعها البيولوجي الاستثنائي، وتقع عند مصب خليج عدن في شمال غرب المحيط الهندي بين إفريقيا والجزيرة العربية. يسكن الجزيرة ما يقارب نحو 60,000 شخص، يعتمدون على الأنشطة شبه الإرتحالية كصيد الأسماك والرعي لتحصيل الغذاء، ويتحدثون بلغة جنوبية عربية حديثة. معظم ما وصل إلينا من معرفة عن تاريخ سُقُطْرَى مأخوذ من كتابات الرحالة الأجانب الذين قدّموا تفاصيل قليلة عن السكان المحليين، ولكن لم يتم بعد البحث المباشر عن الأصول الجغرافية والتاريخ الجيني للشعب السُقُطْري القديم. تقدم هذه الدراسة بيانات الجينوم الكامل لمجموعة من الأفراد وعددهم 39 دفنوا في ستة مواقع على الجزيرة، وعاشوا تقريبا في الفترة ما بين عام 650م حتى عام 1750م. أظهرت الدراسة وجود روابط جينية عالية بين جزيرة سُقُطْرَى ومنطقة حضرموت المعزولة بشكل مماثل في جنوب شبه الجزيرة العربية، مما يجعلها المصدر المرجح لإستيطان سقطرى. يمكن تمثيل الأصول الوراثية للسُقُطْريين في العصور الوسطى كالتالي: ما يقارب من 86% تعود لسكان حضرموت المعاصرين، وما يقارب من 14% تعود لسكان إيران، ونسبة ضئيلة لا تزيد عن 2% تعود لسكان الهند. ومن المرجح أن حركة التجارة المثبتة أثرياً من هذه المناطق أدت إلى هذا الاختلاط الجيني. مقارنة بسكان شبه الجزيرة العربية، فإن تحليل الجينوم للسُقُطْريين في العصور الوسطى يتفق مع عدم وجود اختلاط مع مكوّن إفريقي من جنوب الصحراء الكبرى منذ فترة العصر الجليدي الأخير. ما يميز الأصول القديمة المشتركة لسكان حضرموت وسقطريي العصور الوسطى عن أصول قرنائهم من سكان شبه الجزيرة العربية، بأنها تعود في معظمها إلى الصيادين اللذين استوطنوا الشام منذ فترة العصر الجليدي الأخير (الناطوفيين) أكثر من مزارعي فترة الهولوسين المبكر. تؤكد نتائج هذه الدراسة بأن هجرات المزارعين الأوائل من الشام كان لها تأثير أقل في جنوب شبه الجزيرة العربية وسُقُطْرَى، وتقدم أدلة مقنعة على أنه لم يحدث استبدال كامل للسكان بين العصور الجليدي والهولوسين في جميع أنحاء شبه الجزيرة العربية. كانت سُقُطْرَى في العصور الوسطى تأوي سكانًا قليلين أظهروا ممارسات زواج مختلفة نوعياً عن السُقُطْريين المعاصرين، حيث كانت الزيجات بين الأقارب من الدرجة الأولى تحدث بشكل أقل بكثير مما هو عليه اليوم.

The Arabic version of the Abstract is published as supplied by the authors.

Supplementary Note 2: Permissions and Sampling Protocols

Official permission for the export of skeletal samples to the Reich Lab and subsequent ancient DNA analysis was granted by the Soqotri Governorate and General Organization of Antiquities and Museums (GOAM) on Soqotra. Copies of this permission are available from J. Jansen Van Rensburg upon request. This project also received support from the Environmental Protection Agency on Soqotra (EPA) as well as the Soqotra Heritage Project (SHP) team (**Acknowledgements**).

The choice and suitability of sites for this project was determined in partnership with the local GOAM representative and a local stakeholder, both of whom have good contacts with villagers on the island. All sampling of skeletal material for this project was conducted by J. Jansen Van Rensburg in conjunction with a GOAM representative, a local guide, a driver, and a photographer/ archaeologist and adhered to the following criteria:

- Individuals were chosen for ancient DNA analysis based upon their relevance to archaeological questions, state of preservation, available context information, and geographic location, among other factors;
- No more than one skeletal sample (bone or tooth) was selected per individual to minimize disruption and reduce the impact of destructive analysis;
- Samples were selected to maximize the chance of generating authentic ancient DNA. In most cases, we sampled petrous bone or teeth, which are widely accepted as the most optimal skeletal elements for DNA preservation (Gamba et al., 2014; Hansen et al., 2017; Pinhasi et al., 2015). When these elements were not available, we sampled a dense long bone. We also used appropriate protections (such as frequently-changed nitrile gloves) to minimize modern DNA contamination;
- We were careful to minimize negative impacts on osteological collections, for example by choosing samples with an accessible antimere (opposite-side pair), samples that were already fragmentary, and/or samples that did not exhibit informative morphological or life history traits and did not bear otherwise useful materials such as dental calculus.

All examined skeletal collections and all sampling activity are fully documented in digital and photographic databases which have been shared with the curating institutions.

Details of all samples taken can be found in **Supplementary Data 1**, including those which yielded negative results. Records of all sampling activity can also be obtained by contacting corresponding authors K. Sirak or J. Jansen van Rensburg.

Supplementary Note 3: Archaeological Site Summaries

We provide background on the archaeological sites with individuals who produced usable ancient DNA data. These are the *tafone* sites within the areas of Zaflah, Sayiher Di Hadiboh, Jaher Di Aberho, Jebel Hawari, Fa’ar di Seneqanoh, and Wadi Di Azerho, all of which are in Soqotra, Yemen. Full details of sites and samples tested can be obtained from GOAM and/or relevant publications cited below.

Soqotra Heritage Project (SHP) refers to a site indexing system for Soqotra. Latitudes and longitudes are approximate and have been rounded to the nearest thousandth of a degree; more detailed site locations may be obtained from the curating institutions and/or relevant publications. Elevation is given in meters above sea level (m.a.s.l.). Dates in this section are presented as uncalibrated years before present (BP), and as calibrated years before present (calBP). Calibrations use 95.4% intervals from OxCal v4.4.2 (Bronk Ramsey & Lee, 2013) and the IntCal20 northern hemisphere curve (Reimer et al., 2020).

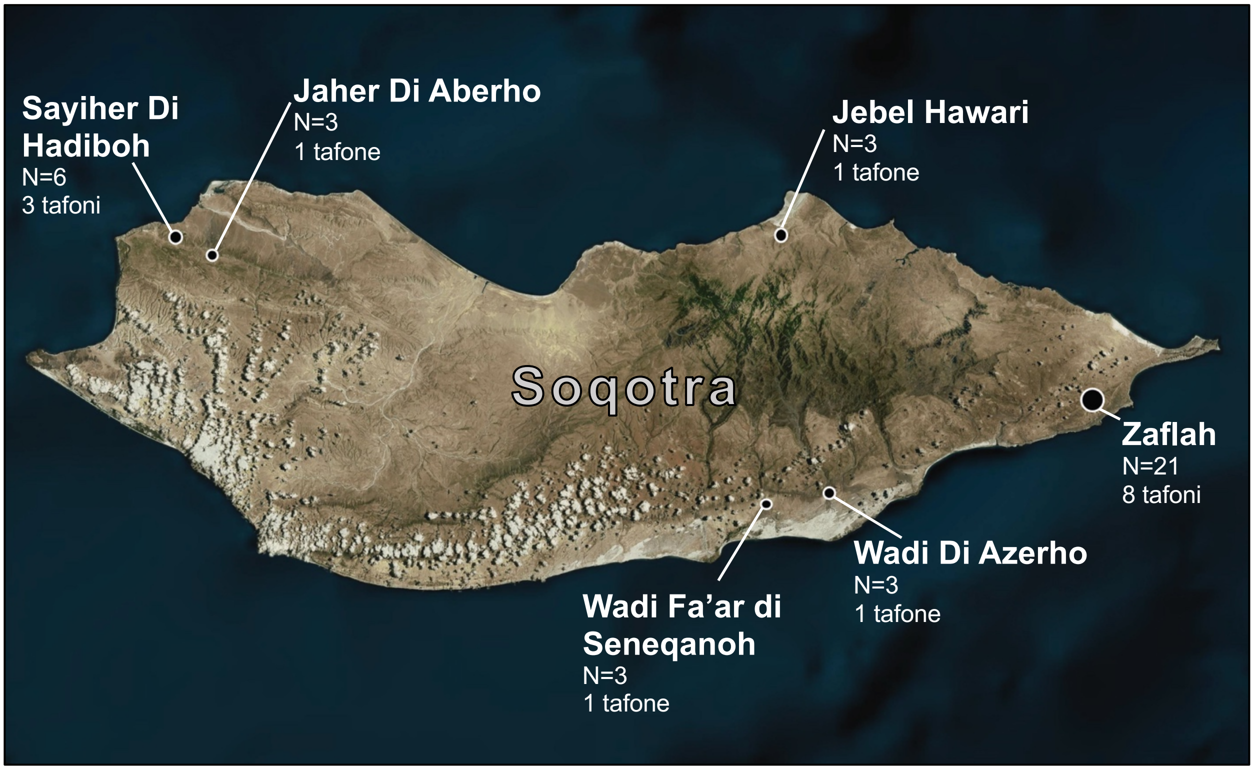

Figure S1. Map of Soqotra with sites, number of tafone at each site, and number of individuals from each site marked. Basemap is from Google Earth (Google Earth, Data SIO, NOAA, U.S. Navy, NGA, GEBCO, Image Landsat / Copernicus).

Jebel Hawari

<u>SHP364 (Tafone A)</u>
Latitude: 12.667016, Longitude: 54.068283. Elevation: 261 m.a.s.l.
SHP364 is a tafone located on a cliff face on the southern half of Jebel Hawari. Access to the tafone is only possible by traversing a thin ledge along the cliff, making access to the site perilous. The tafone measures approximately 3.2 m in length and 1.7 m in width at the entrance, tapering to 0.20 m at end. The dry-stone walled entrance has been partially destroyed by looters, who pushed the walling into the tafone crushing some of the skeletal remains before looting the tomb. The commingled skeletal remains recorded within the tomb included six skulls, five of which were located at the entrance suggesting that the remains were

interred with the heads facing the entrance. We collected three cochleae samples from the skeletal remains of three individuals at the entrance (SHP364/SK4/1, SHP364/SK6/2, SHP364/SK2/3).

Zaflah

SHP217 (Tafone B)

Latitude: 12.50936667, Longitude: 54.43145. Elevation: 445 m.a.s.l.

SHP217 is a tafone situated at the base of a cliff that overlooks a large plain, below a series of tafone where skeletal remains were recorded. The dry-stone wall at the entrance has been almost destroyed by looters. The tafone measures approximately 4.2 meters in length and 2.8 meters in width. The commingled skeletal remains are spread throughout the western half of the tafone and consist of six skulls and approximately 10 long bones. We collected samples from two teeth (SHP217/SK1/1, SHP217/SK4/2).

SHP354 (Tafone C)

Latitude: 12.50936667, Longitude: 54.43145. Elevation: 447 m.a.s.l.

SHP354 is a tafone situated halfway up a cliff that overlooks a large plain. Access to the site is only possible by climbing a narrow fissure that runs up the cliff face. The dry-stone wall entrance is made of large slabs of rocks that has been partly destroyed by looters entering the cave. The tafone measures approximately 2.6 meters in length and 1.3 meters in width. The commingled skeletal remains are mostly within the central part of the tafone, although it is likely more remains are buried in the rubble from the collapse of the dry-stone wall. We collected samples from three teeth (SHP354/SK1/3, SHP354/SK2/2, SHP354/SK3/1).

SHP353 (Tafone D)

Latitude: 12.50936667, Longitude: 54.43145. Elevation: 440 m.a.s.l.

SHP353 is a tafone situated at the base of a cliff that overlooks a large plain, below a series of tafone where skeletal remains were recorded. The dry-stone wall at the entrance has been partly destroyed by looters entering the cave. The tafone measures approximately 3.8 meters in length and 1.3 meters in width and has small alcove in the southern edge of the tafone. The commingled skeletal remains are mostly spread along the eastern edge, with one skull found in the southern alcove. We collected samples from two teeth (SHP353/SK3/1, SHP353/SK4/2).

SHP355 (Tafone E)

Latitude: 12.50936667, Longitude: 54.43145. Elevation: 447 m.a.s.l.

SHP355 is a tafone situated halfway up a cliff that overlooks a large plain. Access to the site is only possible by climbing a narrow fissure that runs up the cliff face. Dry-stone walling is built part way up the fissure to the entrance of the tafone, where looters have pushed the walling into the tafone. The tafone measures approximately 3.1 meters in length and 1.5 meters in width. The commingled skeletal remains are mostly found at the back of the tafone, although it is likely more remains are buried in the rubble from the collapse of the dry-stone wall. We collected samples from three teeth (SHP355/SK3/3, SHP355/SK1/1, SHP355/SK2/2).

SHP216 (Tafone F)

Latitude: 12.50936667, Longitude: 54.43145. Elevation: 443 m.a.s.l.

SHP216 is a tafone situated half-way up a cliff face that overlooks a large plain closely associated with a series of other tafone, where skeletal remains were recorded. The dry-stone walling is made up of large flat rocks that reach to the ceiling along the edges of the tafone. Looters have removed several courses of the central dry-stone wall to gain access. The tafone measures approximately 5.2 meters in length and 3.1 meters in width. The commingled skeletal remains are found along the eastern edge of the tafone, concentrated within a central alcove and at the rear. The skeletal remains consist of 10 skulls and two long bones. We collected samples from five teeth (SHP216/SK3/3, SHP216/SK1/1, SHP216/SK2/2, SHP216/SK5/4, SHP216/SK6/5).

SHP356 (Tafone G)

Latitude: 12.50936667, Longitude: 54.43145. Elevation: 443 m.a.s.l.

SHP356 is a tafone situated halfway up a cliff that overlooks a large plain. Access to the site is only possible by climbing a narrow fissure that runs up the cliff face. The dry-stone wall entrance is made of large and small rocks that has been partly destroyed by looters entering the cave. The tafone measures approximately

3.3 meters in length and 2.1 meters in width. The commingled skeletal remains are mostly spread in the south-eastern alcove, although it is likely more remains are buried in the rubble from the collapse of the dry-stone wall. We collected samples from three teeth (SHP356/SK3/3, SHP356/SK1/1, SHP356/SK2/2).

<u>SHP362 (Tafone H)</u>
Latitude: 12.50936667, Longitude: 54.43145. Elevation: 438 m.a.s.l.
SHP362 is a tafone situated halfway up a cliff that overlooks a large plain. Access to the site is only possible by climbing up a narrow fissure that runs up the cliff face to the side of the tafone. The dry-stone wall entrance is made of large slabs of rock that has been partly destroyed by looters entering the cave. The tafone measures approximately 3.1 meters in length and 1.2 meters in width. The commingled skeletal remains are located throughout the tafone, and it is likely more are buried beneath the dry-stone walling that was pushed into the tafone by the looters. We collected a sample from one tooth (SHP362/SK1/1).

<u>SHP359 (Tafone I)</u>
Latitude: 12.50936667, Longitude: 54.43145. Elevation: 437 m.a.s.l.
SHP359 is a tafone situated halfway up a cliff that overlooks a large plain. Access to the site is only possible by climbing a narrow fissure that runs up the cliff face. The dry-stone wall entrance is made of large slabs of rock that has been partly destroyed by looters entering the cave. The tafone measures approximately 2.5 meters in length and 0.9 meters in width. The commingled skeletal remains are located throughout the tafone. We collected samples from one tooth (SHP359/SK1/1), and one cochlea (SHP359/SK2/2).

Wadi Di Azerho

<u>SHP039 (Tafone J)</u>
Latitude: 12.422533, Longitude: 54.123583. Elevation: 103 m.a.s.l.
SHP039 is a large tafone situated approximately 20 m above the floor of the valley on a large cliff. Access is only possible by climbing along a split in the rock to the east of the tafone. The north-facing entrance of the tafone is approximately 5.6 m wide and extends northwards for approximately 3.4 m. The entrance was once closed with a recessed dry-stone wall that in the west forms a room with a north-facing entrance. In the east, the wall has been destroyed by looters, and little remains of the internal built structure. Commingled skeletal remains are found throughout the tafone that due to looting have been disturbed. Within the western room the skeletal remains are mostly found near the entrance in the south. These comprised of 11 skulls and approximately 16 long bones. In the eastern area the skeletal remains have been pushed towards the entrance by looters and the majority of the 18 skulls recorded were found at the entrance or push against the remnants of the dry-stone walling in the southeast. We collected two teeth (SHP039/SK19/2, SHP039/SK23/3) and one cochlea (SHP039/SK26/1).

<u>SHP040 (No working data were generated from the individuals in this tafone)</u>
Latitude: 12.422533, Longitude: 54.123583. Elevation: 73 m.a.s.l.
SHP040 is a tafone situated approximately five meters above the floor of the wadi within a large boulder. The dry-stone walled entrance consists of large and small rocks that have almost all been removed by looters. The tafone is approximately 2.1 meters in width and 2.4 m in length. The commingled skeletal remains are situated at the entrance in the south of the tafone and consist of heavily disturbed remnants of approximately three long bones. We collected two long bone samples (SHP040/SK2/1, SHP040/SK3/2).

<u>SHP0370 (No working data were generated from the individuals in this tafone)</u>
Latitude: 12.422533, Longitude: 54.123583. Elevation: 98 m.a.s.l.
SHP0370 is a tafone situated on the base of a large boulder on the floor of a wadi. The dry-stone walling is approximately five courses high, reaching the ceiling of the tafone in the south. The northwestern half of the wall has fallen outwards. The tafone measures approximately 3.9 meters in length and 1.6 meters in width. The commingled skeletal remains are spread throughout the walled area and consist of approximately 19 long bones and three skulls. We collected samples from three long bones (SHP370/SK2/1, SHP370/SK3/2, SHP370/SK4/3).

<u>SHP0372 (No working data were generated from the individuals in this tafone)</u>

Latitude: 12.422533, Longitude: 54.123583. Elevation: 96 m.a.s.l.
SHP0372 is a tafone situated on the base of a large boulder approximately two meters above the wadi floor. The dry-stone walling is mostly intact and extends to the ceiling of the tafone, apart from the south where the walling has been pushed inwards by looters. The tafone measures approximately 3.2 meters in length and 1.2 meters in width. The commingled skeletal remains are found in the northwest and consist of approximately 11 long bones, a pelvis fragment and three skulls. We collected samples from two long bones (SHP372/SK3/1, SHP372/SK4/2).

Wadi Fa'ar di Seneqanoh

SHP0368 (Tafone K)
Latitude: 12.422533, Longitude: 54.123583. Elevation: 98 m.a.s.l.
SHP0368 is a tafone situated at the base of a large boulder on the floor of the wadi. The dry-stone walled entrance consists of large and small stones that have been destroyed by looters. The tafone measures approximately 4.2 meters in length and 3.2 meters in width. The commingled skeletal remains have been spread throughout the interior by looters and consist of eight skulls and approximately 3 long bones. We collected a tooth (SHP368/SK6/3), and two cochleae (SHP368/SK2/2, SHP368/SK4/1).

Jaher Di Aberho

SHP375 (Tafone L)
Latitude: 12.651266, Longitude: 53.466483. Elevation: 189 m.a.s.l.
SHP375 is a tafone located at the top of escarpment at the base of a large boulder. The dry-stone walled entrance is composed of large and small rocks that have been partially removed by looters. The tafone runs along the side of the boulder in a north-south direction for approximately 4.2 m and extends west under the boulder for approximately 2.3 m. The western edge forms two alcoves within which commingled skeletal remains were found. The northernmost alcove was partly walled off with dry-stone walling, approximately five courses high. The skeletal remains recorded in this alcove included 11 skulls and numerous disarticulated remains. The southernmost alcove was walled with dry-stone walling, approximately three courses high, that had collapsed into the alcove and crushed several skulls. The skeletal remains recorded included seven skulls and three long bones. We collected two cochleae from the southern alcove (SHP375/SK4/1), SHP375/SK7/2), and two tooth samples from the northern alcove (SHP375/SK10/3), SHP375/SK17/4). Genetic analysis revealed that the tooth samples were from the same individual (labeled I20718 in this work).

Sayiher Di Hadiboh

SHP376 (Tafone M)
Latitude: 12.665383, Longitude: 53.444983. Elevation: 98 m.a.s.l.
SHP376 is a tafone situated at the base of a rock ridge on a terraced platform closely associated with a series of other tafone, where skeletal remains were recorded, SHP378, SHP381. The dry-stone walled entrance consists of large and small rocks that have partially collapsed. The tafone extends under the rock in a southeasterly direction for approximately 3.4 m and is approximately 2.4 m in width. The commingled skeletal remains are all located along the western half of the tafone, and consist of three skulls, a pelvic bone and several long bones and ribs. We collected a cochlea (SHP376/SK1/1) and a tooth sample (SHP376/SK2/2).

SHP381 (Tafone N)
Latitude: 12.665383, Longitude: 53.444983. Elevation: 101 m.a.s.l.
SHP381 is a tafone situated at the base of a rock ridge on a terraced platform closely associated with a series of other tafone, where skeletal remains were recorded, SHP376, SHP378. The entrance to the tafone is located at the edge of the base of a rock ridge. The dry-stone walled entrance consists of large and small

rocks, the uppermost of which have been pushed into the tafone by looters. The tafone extends under the rock in a southwesterly direction for approximately 2.3 m and is approximately 2.8 m in width at its widest part. The commingled skeletal remains are situated along the western most area of the tafone and consist of five skulls and six long bones. We collected two teeth (SHP381/SK1/1), SHP381/SK3/2).

SHP378 (Tafone O)
Latitude: 12.665383, Longitude: 53.444983. Elevation: 108 m.a.s.l.
SHP378 is a tafone situated at the base of a rock ridge on a terraced platform closely associated with a series of other tafone, where skeletal remains were recorded, SHP376, SHP381. The entrance to the tafone is located behind a large boulder making the site difficult to find. The dry-stone walled entrance consists of large and small rocks, the uppermost of which have been pushed into the tafone by looters. The tafone extends under the rock in a southeasterly direction for approximately 4.2 m and is approximately 1.8 m in width. The commingled skeletal remains are located midway into the tafone and consist of four skulls and four long bones. We collected two teeth (SHP378/SK11/2, SHP378/SK2/1) and one cochlea (SHP378/SK4/3).

Supplementary Note 4: PCA setup and dataset information

With *smartpca v.18200*, we attempted to build a PCA that would allow differentiation among African ancestry, Near Eastern ancestry, and Central /South Asian ancestry. We used the parameters 'lsqproject: YES', 'newshrink: YES', 'numoutlieriter: 2', and 'hiprec: YES' and carried out PCA with the following set of 1198 individuals from 63 African, Eurasian, and Pacific populations genotyped on the Affymetrix Human Origins SNP array (referred to as the "Human Origins (HO) dataset"). The population labels of the individuals used to construct the axes with the sample size and corresponding reference(s) are:

Pop	N	Reference(s)	Pop	N	Reference(s)
Armenian	10	1	Lebanese_Christian	9	5
Balochi	20	2	Lebanese_Muslim	11	5
Belarusian	10	1	Lezgin	9	1
Brahui	21	2	Libyan	5	5
Bulgarian	10	1	Lithuanian	10	1
Burusho	23	2	Makrani	20	2
Croatian	10	1	Maltese	8	1
Cypriot	8	1	Muslim_Jat	4	6
Druze	39	2	Ossetian	14	1
Egyptian	18	1	Pathan	17	2
English	10	1	Romanian	10	5
Ezid	8	3	Russian	71	3,2
Finnish	7	1	Sardinian	25	2
French	61	1	Saudi	8	1
Georgian	23	1	Shia_Iranian_Hyderabad	4	6
Hungarian	20	1	Sicilian	11	1
IBS_CanaryIslands	2	1	Sikh_Jatt	41	6
India_Non_Zoroastrian_Hindu	11	4	Sindhi_Pakistan	14	2
India_Zoroastrian	13	4	Spanish	172	7,1
Iran_Non_Zoroastrian	17	4	Tajik	31	3
Iran_Zoroastrian	24	4	Tamta	1	6
Iranian	38	1,5	Turkish	50	1
Iranian_Bandari	8	5	Turkmen	6	1
Italian_Sardinian	2	2	Ulladan	17	6
Jain	3	6	Uzbek	27	3
Jordanian	9	1	Yemeni_Desert	16	8
Juang	26	6	Yemeni_Desert2	15	8
Kalash	17	2	BedouinB	19	2
Kamboj	31	6	Kankanaey	10	9
Kumyk	8	1	Nasioi	10	2
Kurd	8	3	Baining_Marabu	10	9
Lebanese	8	1			

Reference Key:
1: Lazaridis et al., 2014
2: Patterson et al., 2012
3: Jeong et al., 2019
4: Broushaki et al., 2016
5: Lazaridis et al., 2016
6: Nakatsuka et al., 2017
7: Biagini et al., 2019
8: Vyas et al., 2017
9: Skoglund et al., 2016

All ancient individuals and additional present-day groups were then projected onto the first two principal components computed using the above dataset. The option 'newshrink: YES' remaps the points for the individuals used to generate the PCA onto the positions where they would be expected to fall if they had been projected, thereby allowing the co-visualization of projected and nonprojected individuals. Along with the newly-reported ancient individuals from Soqotra (**Supplementary Data 2**), we plotted the following ancient and modern groups:

Pop	Classification on PCA	Anc/Mod	Reference
Ethiopia_Mursi	Horn of Africa Nilotic	Modern	López et al., 2021
Ethiopia_Tigraway	Horn of Africa Afroasiatic Semitic	Modern	López et al., 2021
Ethiopia_Ari_Cultivator	Horn of Africa Afroasiatic Omotic	Modern	López et al., 2021
Egyptian*	Egyptian	Modern	Lazaridis et al., 2014
Algerian	Algerian	Modern	Lazaridis et al., 2014
Jordanian*	Jordanian	Modern	Lazaridis et al., 2014

Lebanese_Christian*	Lebanese_Christian	Modern	Lazaridis et al., 2016
Druze*	Druze	Modern	Patterson et al., 2012
BedouinB*	BedouinB	Modern	Patterson et al., 2012
Saudi*	Saudi	Modern	Lazaridis et al., 2014
Iranian*	Iranian	Modern	Lazaridis et al., 2014; Lazaridis et al., 2016
Georgian*	Georgian	Modern	Jeong et al., 2019
Kalash*	Kalash (Cline-ANI)	Modern	Patterson et al., 2012
Rajput	Rajput (Cline-ASI+ANI)	Modern	Nakatsuka et al., 2017
Mala	Mala (Cline-ASI)	Modern	Lazaridis et al., 2014
Ulladan*	Ulladan	Modern	Nakatsuka et al., 2017
Greek	Greek	Modern	Lazaridis et al. 2014
Sardinian	Sardinian	Modern	Patterson et al., 2012
Lithuanian	Lithuanian	Modern	Lazaridis et al., 2014
Yemeni_Desert*	Yemeni_Desert	Modern	Vyas et al., 2017
Yemeni_Desert2*	Yemeni_Desert2	Modern	Vyas et al., 2017
Yemeni_Highlands	Yemeni_Highlands	Modern	Vyas et al., 2017
Yemeni_Highlands_Raymah	Yemeni_Highlands_Raymah	Modern	Vyas et al., 2017
Yemeni_Northwest	Yemeni_Northwest	Modern	Vyas et al., 2017
Jew_Yemenite	Jew_Yemenite	Modern	Lazaridis et al., 2014
Yemeni	Yemeni	Modern	Lazaridis et al., 2014
Kenya_PastoralNeolithic	Kenya PN	Ancient	Prendergast et al., 2019
Kenya_Manda_Swahili	Kenya Manda	Ancient	Brielle et al., 2023
Kenya_Mtwapa_Swahili	Kenya Mtwapa	Ancient	Brielle et al., 2023
Iran_GanjDareh_Neolithic	Iran Ganj Dareh N	Ancient	Lazaridis et al., 2016; Narasimhan et al., 2019
Iran_TepeHissar_Chalcolithic	Iran Tepe Hissar ChL	Ancient	Narasimhan et al., 2019
Turkey_Barcin_Neolithic	TUR Barcin N	Ancient	Mathieson et al., 2015; Lazaridis, Alpaslan-Roodenberg, et al., 2022b
Israel_Natufian_EpiPaleolithic	ISR Natufian EpiP	Ancient	Lazaridis et al., 2016; Lazaridis, Alpaslan-Roodenberg et al., 2022a, 2022b
Levant_PPNB_Neolithic	Levant PPNB	Ancient	Lazaridis et al., 2016; Narasimhan et al., 2019; Lazaridis, Alpaslan-Roodenberg et al., 2022a, 2022b
Levant_Chalcolithic	Levant ChL	Ancient	Harney et al., 2018
Meggido_BronzeAge	Levant BA	Ancient	Agranat-Tamir et al., 2020
Ancient_Egyptian	Egypt Pre-Ptolemaic/Ptolemaic	Ancient	Schuenemann et al., 2017

Asterisk (*) denotes that population was used to compute axes.

Using the HO array dataset for PCA as well as some other analyses enables us to include genotype data for multiple present-day people from multiple parts of Yemen, including eight Yemenite Jews (‘Jew_Yemenite’) and six Yemeni individuals described in (Kivisild et al., 2004) as individuals of Yemeni matrilineal descent living in Kuwait (‘Yemeni’; genomic data first published in (Lazaridis et al., 2014), as well as 89 individuals from Yemen who were grouped first by region and second by ancestry, reported in (Vyas et al., 2017): ‘Yemeni_Desert’ (16 individuals from Hadramawt, Mahra, and Shabwah governates who had very little or no sub-Saharan African-related ancestry, ‘Yemeni_Desert2’ (15 individuals from Hadramawt, Mahra, and Shabwah governates who had substantial proportions of sub-Saharan African-related ancestry), ‘Yemeni_Highlands’ (29 individuals from Ad Dali, Al Bayda, Al Mahwit, Amanata Al Asimah, Dhamar, Ibb, and Sana’a governates), ‘Yemeni_Highlands_Raymah’ (one individual from the Raymah governate who had substantially more sub-Saharan African-related ancestry than others from this region), and ‘Yemeni_Northwest’ (28 individuals from the Al Jawf, Amran, Ma’rib, and Sa’dah governates). With this dataset, we are also able to use data from a Saudi population from Saudi Arabia and the BedouinB population that we refer to in the main manuscript as “Arabian-related.” Although these Bedouin individuals were sampled from the area of present-day Israel, they are from a culture that historically inhabited the desert regions of the Arabian Peninsula, the Levant, and North Africa and are more genetically similar to populations from the Arabian Peninsula than other groups in the Levant (Almarri et al., 2021).

Supplementary Note 5: qpWave *Modeling*

Testing for cladality among the medieval Soqotri individuals with the 1240k dataset

For tests of cladality between every pair of medieval Soqotri individuals of sufficient coverage (>0.01x coverage, n=32), we used *qpWave* v.1540. Here we use the "1240k dataset" (referring to the dataset leveraging the full set of sites targeted by the 1240k in-solution enrichment reagent) to maximize the amount of data for this analysis. We used 'allsnps: YES' which calls *qpfstats* to calculate the relevant f-statistics, set Han.SDG as the base population ('basepop: Han.SDG') for all *qpfstats* calculations (https://github.com/DReichLab/AdmixTools/blob/master/qpfs.pdf), and set 'inbreed: NO' but also added the 'inbreedlistname' parameter so that any population with more than one individual would instead be analyzed as if we set 'inbreed: YES'. For the reference set (sometimes referred to as the "right" populations), we used a reference set of diverse present-day groups with diploid data to maximize the resolution of our analysis: Altaian.DG, Iranian.DG, Ami.DG, Kalash.DG, Brahmin.DG, Onge.DG, Igorot.DG, French.DG, Mozabite.DG, BedouinB.DG, Jew_Yemenite.DG, Druze.DG, Dinka.DG, Agaw.DG, and BantuKenya.DG.

Consistent with PCA, I21109 stands out as a genetic outlier, forming a clade with only five other Soqotri individuals at a threshold of p>0.05 (See **Figure S1** in main text and **Supplementary Data 4).**

We then tested for cladality between I21109 and a pool of the other Soqotri individuals (n=27 after also excluding one individual from each of four first-degree relative pairs) and obtain results that are not consistent with cladality (p=0.003 for a rank 0 model). Based on these results, we removed I21109 from the Soqotri group (Yemen_Soqotri) in subsequent analyses. The poor preservation of DNA from I21109 – only 2.6% of targeted positions yielding data – precluded high resolution analyses, and thus we focus on the high-quality data we obtained from the 'Yemen_Soqotri' cluster.

Supplementary Note 6:* qpAdm *Modeling

Ancestry modeling with qpAdm

The use of proxy groups for the source populations in *qpAdm* modeling does not imply an assumption that they are directly ancestral to the true source populations of the individuals we analyze. Rather, it implies a weaker assumption that they are descended without admixture from populations that truly are truly ancestral to the sources. *qpAdm* also assumes that no population in the reference set shares genetic drift with the modeled population beyond what is shared in the proxy sources.

Distal* qpAdm *modeling of Yemen_Soqotri

We used the *qpAdm* framework to model the ancestry of Yemen_Soqotri in terms of proxy sources that lived deep in time. As in *qpWave* analyses, we used 'allsnps: YES' which calls *qpfstats* to compute f-statistics. We set Han.SDG as the base population ('basepop: Han.SDG') for all *qpfstats* calculations (https://github.com/DReichLab/AdmixTools/blob/master/qpfs.pdf) and 'inbreed: NO', but also added the 'inbreedlistname' parameter so any population with more than one individual would be analyzed as if we set 'inbreed: YES'.

With a reference set of Mbuti.DG, Papuan.DG, Russia_Ust_Ishim_HG_published.DG, Russia_MA1_HG.SG, CHG.SG, Morocco_EN.SG, Turkey_Boncuklu_N.SG and tested models for the ancestry of Yemen_Soqotri and present-day groups from the Arabian Peninsula as well as Bedouins (who are documented to be genetically more Arabian-related than Levant-related, see **Supplementary Note 4**). As sources, we used ISR_Natufian_EpiP (Natufian hunter-gatherers) or Levant_PPNB (Neolithic Levantine farmers), TUR_Barcin_N (Neolithic Anatolian farmers), Iran_N (Neolithic Iranian herders), all groups which have been shown in previous work to be useful surrogates for ancestry found in Eurasians (Lazaridis et al., 2016; Lazaridis, Alpaslan-Roodenberg et al., 2022a). We also included Ethiopia_4500BP as an African-related source used to represent unadmixed African-related ancestry. While the actual source of African-related ancestry is likely to be only extremely distantly related to this ancient Ethiopian forager, Ethiopia_4500BP serves as a good proxy for sub-Saharan African-related admixture. Results are in **Supplementary Data 5** with a subset reported in **Table 1** in the main manuscript.

We find that the majority of present-day Arabian or Arabian-related groups can be modeled as having three components of ancestry: ancient Levantine/Anatolian-related, ancient Iranian-related, and African-related. Neolithic Levantine and Anatolian people have been shown to share relatedness along a cline (i.e., they are not fully independent ancestry sources (Lazaridis, Alpaslan-Roodenberg, et al., 2022a, 2022b), with Natufians documented as being "more Levantine" along this cline. The Levantine/Anatolian ancestry in our models can, in the majority of cases, be contributed by either Levant_PPNB alone or by a dual source of ISR_Natufian_EpiP and TUR_Barcin_N, as these two populations both contribute genetically to Levant_PPNB which falls intermediate between them on the Levantine/Anatolian ancestry cline (Lazaridis et al. 2016; Lazaridis, Alpaslan-Roodenberg et al., 2022a, 2022b). Consistent with previous work, we model Levant_PPNB as having 60.5% ± 6.0% ISR_Natufian_EpiP-related ancestry and 39.5% ± 6.0% TUR_Barcin_N-related ancestry in our framework (p=0.30)).

Of note is that the ancient Soqotri cannot be fit using Levant_PPNB as the sole source of Levantine/Anatolian-related ancestry (that is an ancestry model for the Soqotri gene pool as Levant_PPNB + Iran_N + Ethiopia_4500BP does not fit (p=0.003)). However, this group can be modeled using the dual source of ISR_Natufian_EpiP and TUR_Barcin_N as dual sources for the Levantine/Anatolian-related ancestry, such that a model of the Soqotri gene pool as ISR_Natufian_EpiP + Iran_N + TUR_Barcin_N + Ethiopia_4500BP fits well (p=0.13) (data in **Table 1** of the main manuscript). This suggests that the Levantine/Anatolian component in the Soqotri gene pool is somewhat "more Levantine" than Levant_PPNB is itself (see paragraph above for ancestry estimates of Levant_PPNB). A similar pattern is seen for Yemeni_Desert and Yemeni_Desert2, present-day groups shown by other analyses to be share genetic drift with the Soqotri (**Table 1;** of note is that Yemeni_Desert2 is differentiated from the Soqotri and Yemeni_Desert because of a high proportion of sub-Saharan African-related ancestry).

We estimate the proportion of ancestry contributed specifically by ISR_Natufian_EpiP in models that use ISR_Natufian_EpiP and TUR_Barcin_N as dual Levantine/Anatolian-related ancestry sources and present these data in **Table 1**. The three groups with the highest (>64%) amount of ancestry associated with

ISR_Natufian_EpiP are Yemen_Soqotri, Yemeni_Desert, and Yemeni_Desert2, while all other Arabian and Arabian-related groups have <59% of the total Levantine/Anatolian-related ancestry modeled by ISR_Natufian_EpiP. As Levant_PPNB is modeled as having ~61% ISR_Natufian_EpiP-related ancestry, our results are consistent with the Levantine/Anatolian-related ancestry in the Yemeni_Desert groups as well as in Yemen_Soqotri being less Anatolian-related and more Levantine-related than that in Levant_PPNB.

The finding of a reduced amount of TUR_Barcin_N-related ancestry in the Soqotri relative to Levant_PPNB and present-day Arabian and Arabian-related groups suggests that the mixture events that introduced Levantine farmer-related ancestry to the Arabian Peninsula did not impact the Yemeni_Desert group or the ancient people of Soqotra to the same extent as other places in Arabia. Additional ancient DNA data from mainland Arabia will help to further elucidate this phenomenon in greater detail, but these findings are consistent with a lower level of displacement of Natufian hunter-gatherer-related ancestry in the Hadramawt area and Soqotra. Plausibly, this reflects the relative isolation of these areas.

We then attempted to fit four-source models for the Arabian and Arabian-related populations as well as Yemen_Soqotri with both Levant_PPNB and TUR_Barcin_N as sources (**Table S1**, all data in **Supplementary Data 5**). We find that many present-day Arabian and Arabian-related populations have approximately the same amount of TUR_Barcin_N ancestry as Levant_PPNB (that is, they either need no additional TUR_Barcin_N-related ancestry or a very small proportion of it). In contrast, these models do not fit for Yemen_Soqotri or the Yemeni_Desert and Yemeni_Desert2 groups, who are poorly modeled.

Table S1. Results for four-source models of Yemen_Soqotri and present-day Arabian and Arabian-related populations as Levant_PPNB + Iran_N + TUR_Barcin_N + Ethiopia_4500BP. Models that fit at p>0.05 are in bold and italics and models that fit at p>0.01 are in italics only. Negative ancestry proportions are in red. All data are in **Supplementary Data 5**. "ANC" followed by a number indicates the ancestry proportion represented by the proxy source in the second row. "SE" followed by a number indicates the standard error associated with the ancestry proportion estimate.

TARGET	ANC1	ANC2	ANC3	ANC4	SE1	SE2	SE3	SE4	
	**Levant_ PPNB**	**Iran_ N**	**TUR_ Barcin_ N**	**Ethiopia _ 4500BP**	**Levant _ PPNB**	**Iran_ N**	**TUR_ Barcin_ N**	**Ethiopia _ 4500BP**	**p-value**
BedouinA	0.526	0.330	0.063	0.081	0.060	0.021	0.054	0.007	*0.01898*
BedouinB	0.575	0.303	0.075	0.047	0.068	0.024	0.061	0.008	***0.14129***
Jew_Yemenite	0.565	0.340	0.063	0.033	0.070	0.025	0.062	0.008	***0.11277***
Saudi	0.546	0.361	0.067	0.025	0.067	0.024	0.060	0.008	***0.24517***
Yemen_Soqotri	0.684	0.325	-0.029	0.019	0.084	0.029	0.075	0.009	0.00064
Yemeni	0.425	0.366	0.058	0.150	0.065	0.023	0.058	0.008	***0.08663***
Yemeni_Desert	0.691	0.288	-0.016	0.037	0.087	0.028	0.077	0.009	0.00016
Yemeni_Desert2	0.444	0.286	-0.011	0.281	0.077	0.023	0.066	0.009	0.00113
Yemeni_Highlands	0.604	0.311	0.021	0.063	0.069	0.024	0.060	0.007	0.00547
Yemeni_Highlands_ Raymah	0.553	0.191	0.055	0.201	0.111	0.042	0.099	0.016	***0.14920***
Yemeni_Northwest	0.557	0.318	0.078	0.046	0.065	0.022	0.057	0.007	*0.01806*

Another important question is from where the Iranian-related ancestry in the Soqotri gene pool derives (this ancestry may or may not have arrived at least in part as a package with additional TUR_Barcin_N-related ancestry). One possibility is that it was introduced to Soqotra by people who lived in the Levant after the Neolithic, as previous work has shown that Iranian-related ancestry deriving from the Zagros/Caucasus (along with TUR_Barcin_N-related ancestry) was present in the Levant during the Chalcolithic (Harney et

al. 2018) and increased in proportion throughout the Bronze Age (Agranat-Tamir et al. 2020). To test if our data are consistent with the Soqotri deriving all of their Iranian-related ancestry from a post-Neolithic ancient Levantine source, we test Levant_ChL (people from Peqi'in Cave in the southern Levant dating to the Late Chalcolithic period, Harney et al. 2018) and Levant_BA (people from the site of Megiddo in northern Israel who mostly date to the Middle-to-Late Bronze Age, excluding genetic outliers and one individual from each pair of first-degree relatives) as sources in our *qpAdm* models along with ISR_Natufian_EpiP. We find that fitting models for the Soqotri still require additional Iran_N-related ancestry when Levant_ChL, or Levant_BA are one source. Models that do not include additional Iran_N-related ancestry are a poor fit, indicating that all tested ancient Levantine populations from the Chalcolithic and Bronze Ages have insufficient ancestry of this type to account for that found in the Soqotri gene pool (**Table S2**).

Another possibility is that the location of Soqotra along key maritime trade routes connecting the Arabian Peninsula with India, Persia, and the eastern Mediterranean could have been a vector for introducing additional Iranian-related ancestry into the Soqotri gene pool. To test if our data are consistent with the Soqotri deriving all of their Iranian-related ancestry from a population like eastern Mediterranean traders, we test six groups of Hellenistic, Roman, or Byzantine Era Anatolians (TUR_Aegean_Muğla_Camandras_Dalagöz_Rom, 3 individuals from Camandras and Dalagöz (Aegean, Muğla, Yatağan, Necropole) who lived 27 BCE - 476 CE; TUR_Marmara_İznik_Basilica_RomByz_A, 6 individuals from Basilica (Marmara, Iznik) who lived 350-700 CE; TUR_Marmara_Apollonia_Rom, 3 individuals from Apollonia (Marmara, Bursa, Gölyazı) who lived 100 BCE - 200 CE; Turkey_EarlyByzantine_1, 4 individuals from Zeytinliada (Marmara, Balikesir, Erdek) who lived 600-1000 CE; TUR_SE_Mardin_RomByz, 6 individuals from Aktaş Mevki (Southeast, Mardin, Midyat) and Aktaş Mevki (Southeast, Mardin, Midyat) who lived 500-900CE; Turkey_Southeast_Byzantine, 6 individuals from Oylum Höyük (Southeast, Kilis) and Tilbeşar Höyük (Southeast, Gaziantep) who lived 900-1300 CE; all data published in Lazaridis, Alpaslan-Roodenberg et al., 2022c) as a source in our *qpAdm* models along with ISR_Natufian_EpiP. These individuals are known to have a mix of Anatolian and Iranian ancestry and much lower proportions of EHG- and SRB_Iron_Gates_HG-related ancestry. Again we find that fitting ancestry models for the Soqotri still require additional Iran_N-related ancestry, indicating that all tested ancient Anatolian populations have insufficient ancestry of this type to account for that found in the Soqotri gene pool (**Table S2**).

Table S2. Two-way and three-way model results for Yemen_Soqotri as a combination of ISR_Natufian_EpiP, a post-Neolithic Levantine or Hellenistic/Roman/Byzantine Anatolian source, and Iran_N. Models that fit at p>0.05 are in bold and italics and models that fit at p>0.01 are in italics only. All data are in **Supplementary Data 6**. "S" followed by a number designates the proxy source population used in the model. "ANC" followed by a number indicates the ancestry proportion represented by the proxy source in the second row. "SE" followed by a number indicates the standard error associated with the ancestry proportion estimate.

TARGET	S1	S2	S3	ANC1	ANC2	ANC3	SE1	SE2	SE3	p-value
Yemen_Soqotri	ISR_Natufian_EpiP	Levant_BA	Iran_N	0.424	0.348	0.228	0.072	0.127	0.071	***0.137403***
Yemen_Soqotri	ISR_Natufian_EpiP	Levant_ChL	Iran_N	0.437	0.252	0.311	0.072	0.097	0.048	***0.103864***
Yemen_Soqotri	ISR_Natufian_EpiP	Levant_BA		0.375	0.625		0.071	0.071		0.000361
Yemen_Soqotri	ISR_Natufian_EpiP	Levant_ChL		0.749	0.251		0.213	0.213		0
Yemen_Soqotri	ISR_Natufian_EpiP	TUR_Aegean_Muğla_Camandras_Dalagöz_Rom	Iran_N	0.507	0.21	0.284	0.055	0.091	0.064	***0.092363***
Yemen_Soqotri	ISR_Natufian_EpiP	TUR_Marmara_İznik_Basilica_RomByz_A	Iran_N	0.508	0.211	0.281	0.049	0.081	0.063	***0.136998***
Yemen_Soqotri	ISR_Natufian_EpiP	TUR_Marmara_Apollonia_Rom	Iran_N	0.477	0.22	0.304	0.067	0.103	0.06	***0.0996***
Yemen_Soqotri	ISR_Natufian_EpiP	Turkey_EarlyByzantine_1	Iran_N	0.5	0.247	0.253	0.051	0.089	0.067	***0.145306***
Yemen_Soqotri	ISR_Natufian_EpiP	Turkey_Southeast_Byzantine	Iran_N	0.503	0.282	0.215	0.052	0.117	0.088	***0.106585***

Yemen_Soqotri	ISR_Natufian_ EpiP	TUR_SE_Mardin_RomByz	Iran_N	0.48	0.331	0.189	0.055	0.125	0.091	***0.122425***
Yemen_Soqotri	ISR_Natufian_ EpiP	TUR_Marmara_İznik_ Basilica_RomByz_A		0.571	0.429		0.062	0.062		0.000055
Yemen_Soqotri	ISR_Natufian_ EpiP	TUR_Aegean_Muğla_ Camandras_Dalagöz_Rom		0.562	0.438		0.077	0.077		0.000029
Yemen_Soqotri	ISR_Natufian_ EpiP	Turkey_EarlyByzantine_1		0.526	0.474		0.062	0.062		0.000586
Yemen_Soqotri	ISR_Natufian_ EpiP	TUR_Marmara_Apollonia _Rom		0.494	0.506		0.241	0.241		0.000003
Yemen_Soqotri	ISR_Natufian_ EpiP	Turkey_Southeast_Byzantine		0.485	0.515		0.053	0.053		0.010375
Yemen_Soqotri	ISR_Natufian_ EpiP	TUR_SE_Mardin_RomByz		0.453	0.547		0.051	0.051		0.021596

Finally, we tested the robustness of a three-source ancestry model in which Yemen_Soqotri can be well-modeled without a distinct sub-Saharan African-related ancestry source. Any ancestry that is “African-related” is, in this model, completely represented by ISR_Natufian_EpiP which previous work has shown to share affinities with ~15,000-year-old Iberomaurusian hunter-gatherers from Morocco (van den Loosdrecht et al. 2018). To test this, we rerun the three-way model (see **Table 1** in the main text), adding a population with most or complete sub-Saharan African-related ancestry added into the reference set (**Supplementary Data 7**). No ancient African-related populations “break” the model (we consider a model to “break” when the addition of a population to the reference set reduces the model p-value to <0.01). That ancient populations with very little or no West Eurasian-related ancestry (for example, Zambia_LSA, Ethiopia_4500BP, Cameroon_SMA, Tanzania_Zanzibar_1300BP, Kenya_LSA, Kenya_Pastoral_IA) can be added to the reference set without reducing the p-value of the model demonstrates it robustness and suggests that the African-related ancestry that may be detected in the Soqotri population can be represented by populations that have been in Asia for at least 14,000 years, as exemplified by the proxy we used for them ISR_Natufian_EpiP. We note that the addition of some present-day groups – mostly those from North Africa and groups with high proportions of West Eurasian-related ancestry from the Horn of Africa (e.g., certain groups from Ethiopia most of whom speak Afro-Asiatic languages) break this model, likely due to common shared ancestry. For example, there is recent shared ancestry between some Afro-Asiatic-speaking groups in the Horn of Africa and Egyptians and West Eurasians.

Proximal* qpAdm *modeling of Yemen_Soqotri: HO dataset

We initially attempted to fit two-source models for Yemen_Soqotri as a mixture of Source 1 (S1) plus Source 2 (S2). We first set Yemeni_Desert as S1 based on the signal of genetic similarity and shared aspects of population history observed in our prior analyses. As S2, we rotated through Eurasian and African populations in the HO dataset (populations listed in **Supplementary Data 16**). We used a small, curated reference set of Ju_hoan_North, Tatar_Siberian, Juang, and Italian_Central.

At p>0.05, only six two-source models fit the data out of 708 tested, and all fit equivalently well (**Table S3**). In these models, Yemeni_Desert fit as a proxy for ~86-93% of the ancestry in the Soqotri gene pool, with the remaining ancestry proxied by a population from the Zagros or a population from the Indian sub-continent with high West Eurasian relatedness and Iranian-related ancestry (**Supplementary Data 16**).

Table S3. All models (out of 708 tested) significant at p>0.05 for two-source models of Yemen_Soqotri as Yemeni_Desert + S2. Models that fit at p>0.05 are in bold and italics and models that fit at p>0.01 are in italics only. Reference set: Ju_hoan_North, Tatar_Siberian, Juang, and Italian_Central. “S” followed by a number designates the proxy source population used in the model. “ANC” followed by a number indicates the ancestry proportion represented by the proxy source in the second row. “SE” indicates the standard error associated with the ancestry proportion estimate.

TARGET	S1	S2	ANC1	ANC2	SE	p-value
Yemen_Soqotri	Yemeni_Desert	India_Zoroastrian	0.897	0.103	0.02	***0.163614***
Yemen_Soqotri	Yemeni_Desert	Iran_Non_Zoroastrian	0.863	0.137	0.032	***0.067946***

Yemen_Soqotri	Yemeni_Desert	Iranian	0.856	0.144	0.033	***0.071039***
Yemen_Soqotri	Yemeni_Desert	Khatri	0.934	0.066	0.013	***0.055858***
Yemen_Soqotri	Yemeni_Desert	Pathan	0.924	0.076	0.016	***0.052417***
Yemen_Soqotri	Yemeni_Desert	Shia_Iranian_Hyderabad	0.88	0.12	0.017	***0.248636***

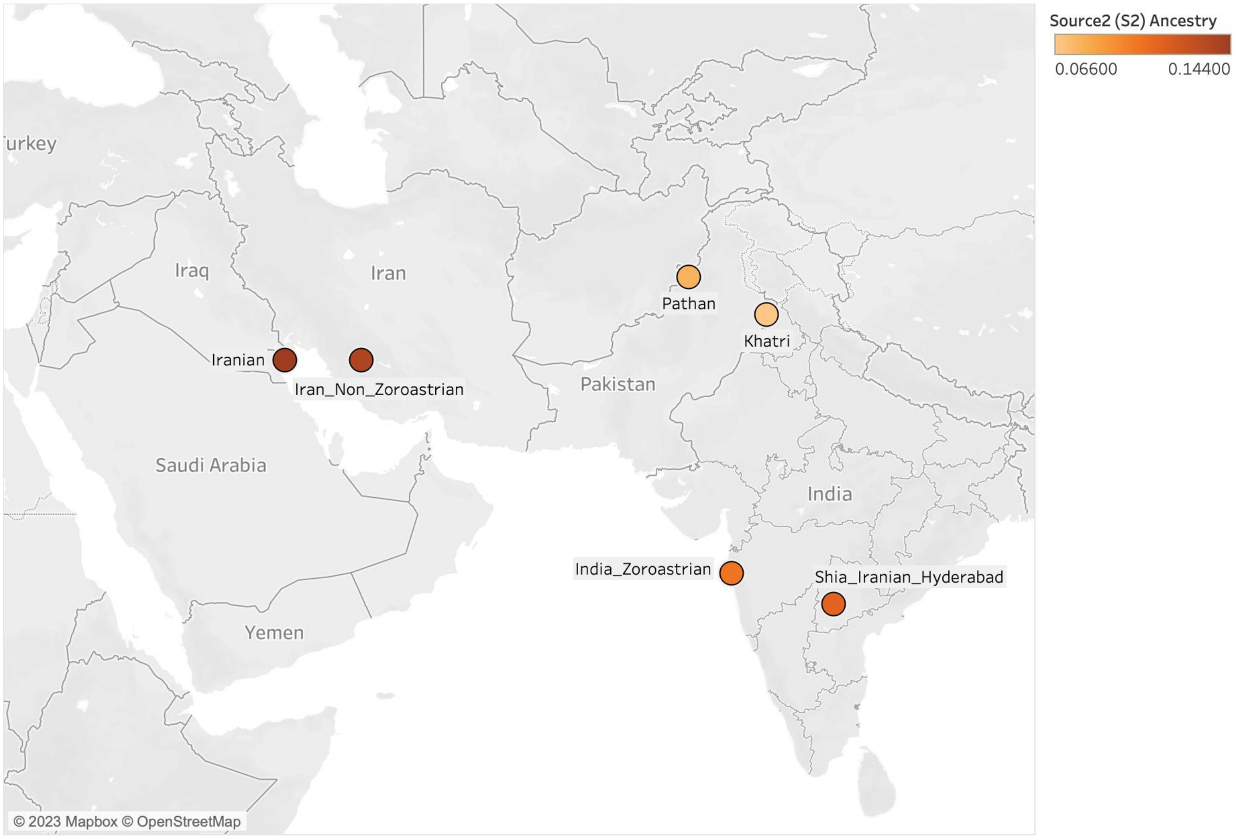

Figure S2. Geographic location of six populations that fit as S2 populations in ancestry models for Yemen_Soqotri. The intensity of the color of the symbol represents the proportion of ancestry contributed by that population in our *qpAdm* model, ranging from 6.6% - 14.4%.

To investigate whether any of the populations that fit as S2 is a significantly better surrogate for the ancestry in Yemen_Soqotri than the others, we used a “model competition” framework based on the idea that the most appropriate surrogate source in the six fitting models should be resilient to the addition of each of the sources of the remaining five models in the reference set (right set) while also being powerful enough to falsify other models (that is, reduce the p-value associated with model fit) when placed in the reference set. We find that no population is able falsify the remaining models, suggesting that none of these populations are significantly closer to the true source population (**Figure S3**).

Population added to reference set

S2	India_Zoroastrian	Iran_Non_Zoroastrian	Iranian	Khatri	Pathan	Shia_Iranian_Hyderabad
India_Zoroastrian		0.13	0.13	0.11	0.11	0.29
Iran_Non_Zoroastrian	0.20		0.03	0.12	0.11	0.21
Iranian	0.15	0.02		0.09	0.08	0.14
Khatri	0.25	0.10	0.11		0.07	0.21
Pathan	0.27	0.09	0.07	0.12		0.25
Shia_Iranian_Hyderabad	0.11	0.03	0.02	0.06	0.05	

Figure S3. Results of model competition framework for six working models in **Table S3**. Numbers in the cells are the p-value for the model using the population in the left-most column as S2 when the population in the top row is added to the reference set. Cells are maroon at $p>0.1$, red at $0.1<p<0.05$, and pink at $0.05<p<0.01$.

We plotted the six populations that fit as S2 on the PCA (**Figure S4;** PCA setup described in **Supplementary Note 4**). The fit of groups such as Khatri and Pathan that fall at the "Ancestral North Indian" end of the Indian cline (groups with ANI ancestry are genetically more similar to West Eurasians including people from the Middle East, Central Asia, and Europe (Reich et al., 2009)), as well as Shia_Iranian_Hyderabad (a group with mixed Iranian and Indian ancestry) as S2 is consistent with a very small amount of Indian-related ancestry in the Soqotri gene pool in addition to a strong signal more closely associated with Iranian-related ancestry.

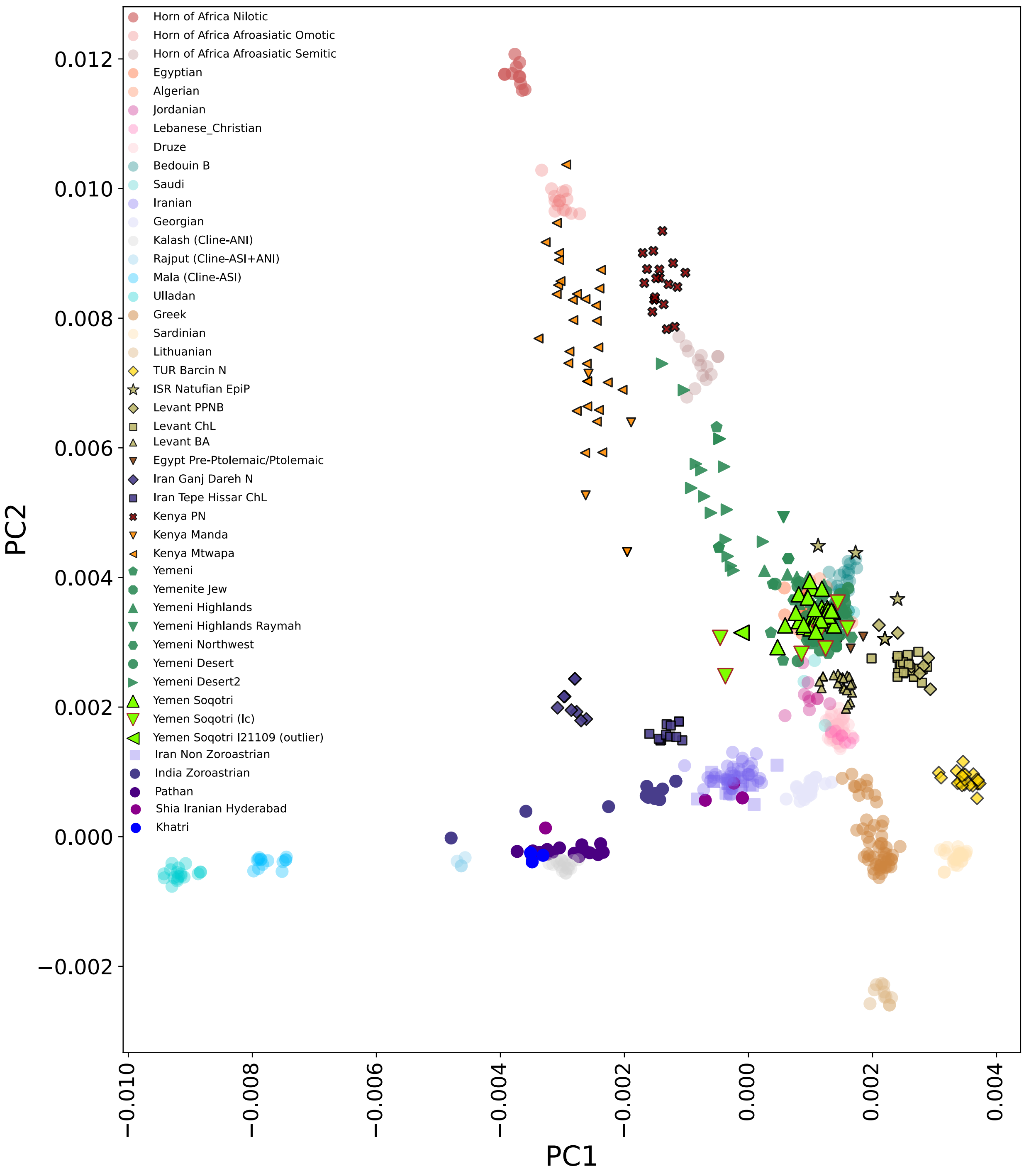

Figure S4. PCA plot (set-up detailed in **Supplementary Note 4**) with six additional populations that fit as S2 in two-source *qpAdm* models plotted.

To approximate the amount of ancestry plausibly deriving from the Indian sub-continent in the Soqotri gene pool, we examined the fit of three-source models, setting Yemeni_Desert as S1, Iranian as S2, and rotating though all African and Eurasian populations in the HO dataset as S3 (**Supplementary Data 17**). We fit three-source models at p>0.05 when populations that make up the Indian cline are S3, although the addition of a third source does not significantly improve or degrade the fit of models suggesting that any Indian

ancestry is at the very limit of our ability to detect it definitively. Specifically examining the fit of models where S3 is an Indian population known to have less ANI (West Eurasian) relatedness and more “Ancestral South Indian” (ASI) ancestry (for example, Bhil, Mala, Madiga, Pulliyar, Ulladan), we find Yemen_Soqotri can be modeled as having as ~0.2-2.3% (ancestry coefficient ± 1 s.e.) Indian-related ancestry in addition to Iranian ancestry (**Table S4**).

Table S4. Models for Yemen_Soqotri as Yemeni_Desert + Iranian + an Indian population with minimal West Eurasian relatedness. Models that fit at p>0.05 are in bold and italics and models that fit at p>0.01 are in italics only. Reference set: Ju_hoan_North, Tatar_Siberian, Juang, and Italian_Central. “S” followed by a number designates the proxy source population used in the model. “ANC” followed by a number indicates the ancestry proportion represented by the proxy source in the second row. “SE” indicates the standard error associated with the ancestry proportion estimate.

TARGET	S1	S2	S3	ANC1	ANC2	ANC3	SE1	SE2	SE3	p-value
Yemen_Soqotri	Yemeni_Desert	Iranian	Bhil	0.863	0.124	0.013	0.038	0.042	0.009	***0.056283***
Yemen_Soqotri	Yemeni_Desert	Iranian	Madiga	0.864	0.122	0.014	0.034	0.039	0.009	***0.056509***
Yemen_Soqotri	Yemeni_Desert	Iranian	Mala	0.863	0.123	0.014	0.036	0.04	0.008	***0.053361***
Yemen_Soqotri	Yemeni_Desert	Iranian	Pulliyar	0.865	0.125	0.01	0.036	0.04	0.008	***0.080944***
Yemen_Soqotri	Yemeni_Desert	Iranian	Ulladan	0.862	0.129	0.009	0.034	0.037	0.007	***0.079371***
Yemen_Soqotri	Yemeni_Desert	Iranian	Bhil	0.863	0.124	0.013	0.038	0.042	0.009	***0.056283***

An interpretation from ancestry modeling is that the Soqotri gene pool has ~86% ancestry well-proxied by Yemeni_Desert, and the remaining ~14% proxied by a group with Iranian-related ancestry that has up to ~2% ancestry that can be traced to the Indian sub-continent.

To test the robusticity of this three-source model, we tried to break it (that is, degrade the fit to p<0.01) by rotating additional populations from the HO dataset into the reference set one by one. We find that 13 populations are able to break the model fit (**Table S5**). Several are from Papua New Guinea or are Austroasiatic-speaking groups from India, suggesting that we do not have the precise proxy Indian-related source in our genotyped data or that there was some gene flow from other Indian or Pacific populations. The majority of populations that broke the three-source model were sub-Saharan Africans with West African-related ancestry, which is suggested by our D-statistics to be present in Yemeni_Desert gene pool (**Supplementary Data 9**).

Table S5. All populations from the HO dataset that reduce the fit of the three-source model for Yemen_Soqotri to p<0.01.

Population added to right	p-value when population is on right
Wambo	0.000606
BantuSA	0.000625
Mandenka	0.000802
Cameroon_Bafut	0.001548
Cameroon_Mbo	0.001742
Htin_Mal	0.003864
Mengen	0.005504
Bajo	0.005637
Mende	0.006198
Biaka	0.008051
Batudi	0.008067
Tshwa	0.008069
Malawi_Tumbuka	0.008585

To test this, we carried out *qpAdm* modeling of Yemeni_Desert, using Yemen_Soqotri as one source and testing the fit of two-source models. We identify only a single fitting two-source model for Yemeni_Desert as 56.1% ± 7.9% Yemen_Soqotri and 43.9% ± 7.9% Yemeni_Highlands (p=0.35), which suggests that some of the same mixture events that impacted Yemeni_Desert also impacted the people of the Yemeni highlands; however, it does not provide information about the origins or affinities of those mixing populations. As no other two-source models fit at p>0.05, we explored the fit of three-source models. As S1 we again fixed Yemen_Soqotri and, based on the results of our D-statistics, we set the West African

genotyped group Cameroon_Mbo as S2. We rotated through all HO populations as S3. We find two well-fitting models (out of 707 tested) that fit Yemeni_Desert as having 79.0% ± 5.0% Yemen_Soqotri-related ancesetry, 3.1% ± 0.6% Cameroon_Mbo-related ancestry, and 17.9% ± 4.5% Sardinian-related ancestry (p=0.12); the other qualitatively similar and quantitatively consistent fitting model replaces Sardinian with Italian_Sardinian, p=0.17). We interpret these results as suggesting that Yemeni_Desert has ~3% additional West African-related ancestry that is not found in the medieval Soqotri gene pool as well as ~18% excess Anatolian-related ancestry.

As a second strategy for attempting to determine whether we can identify and quantify Indian-related ancestry in the medieval Soqotri gene pool, we created artificial populations of varying proportions Iranian-related and Indian-related ancestry. We used Iranian to represent Iranian-related ancestry and Pulliyar to represent Indian ancestry (as in Brielle et al., 2023), and found that it is possible to fit models for Yemen_Soqotri with a proxy S2 population that has 100% Iranian-related ancestry (and therefore 0% Indian-related ancestry) when Yemeni_Desert is S1 (we used the same reference set for these models). However, while there is also no separate detectable Indian component of ancestry, if we use a mixed Iranian-Indian source as S2, we do also produce working models. We find that artificially produced populations of about 80% or more Iranian individuals and the rest Pulliyar individuals provide working models when substituted in place of the completely Iranian source (**Figure S5**). Our results are consistent with a small proportion of Indian-related ancestry in the Soqotri gene pool that is close to or below our limit of definitive detection when Yemeni_Desert is S1, which may be due in part to the presence of varying amounts of Iranian-related ancestry in the gene pools of many South Asian groups.

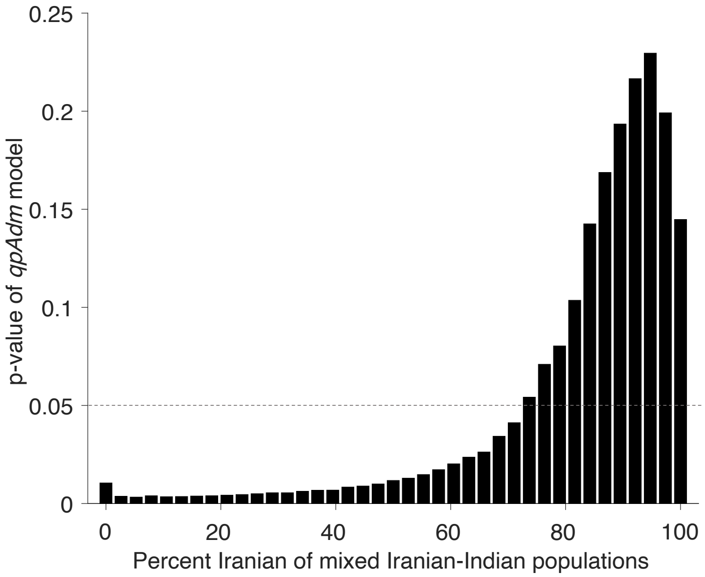

Figure S5. Bar graph showing p-values for a two-way *qpAdm* model with Yemeni_Desert as S1 and an artificially mixed Iranian-Indian source as S2. The x-axis specifies the proportion of Iranian ancestry, with the remaining ancestry made up of the Indian population Pulliyar (a group known to have very little Iranian-related ancestry).

Supplementary Note 7: Outgroup f_3-statistics

We used outgroup f_3-statistics to investigate shared drift between the Soqotri and present-day African and Eurasian populations. A challenge was to select an appropriate outgroup, as the test populations included sub-Saharan Africans as well as groups with no such ancestry. In the main manuscript, we present results using present-day Karitiana from Brazil as an outgroup that allows us to measure shared West Eurasian-related ancestry and illustrates the high amount of shared drift between the medieval Soqotri and populations from the Arabian Peninsula (in particular, Yemeni_Desert). In Figure 1B, we plot the top 100 results when Karitiana is an outgroup, noting that within this group of 100 are some African populations who have high amounts of West Eurasian-related ancestry that is qualitatively similar to the ancestry in the Soqotri gene pool.

Here, we illustrate the outgroup f_3 results we obtain when we use Mbuti as an outgroup (**Figure S6**), which illustrates better the drift shared between the Soqotri and all non-African populations reflecting the Out of Africa bottleneck. With Mbuti as an outgroup, no population from Africa (all of whom have some amount of African-originating ancestry) are in the top 150 results (all data are in **Supplementary Data 8**).

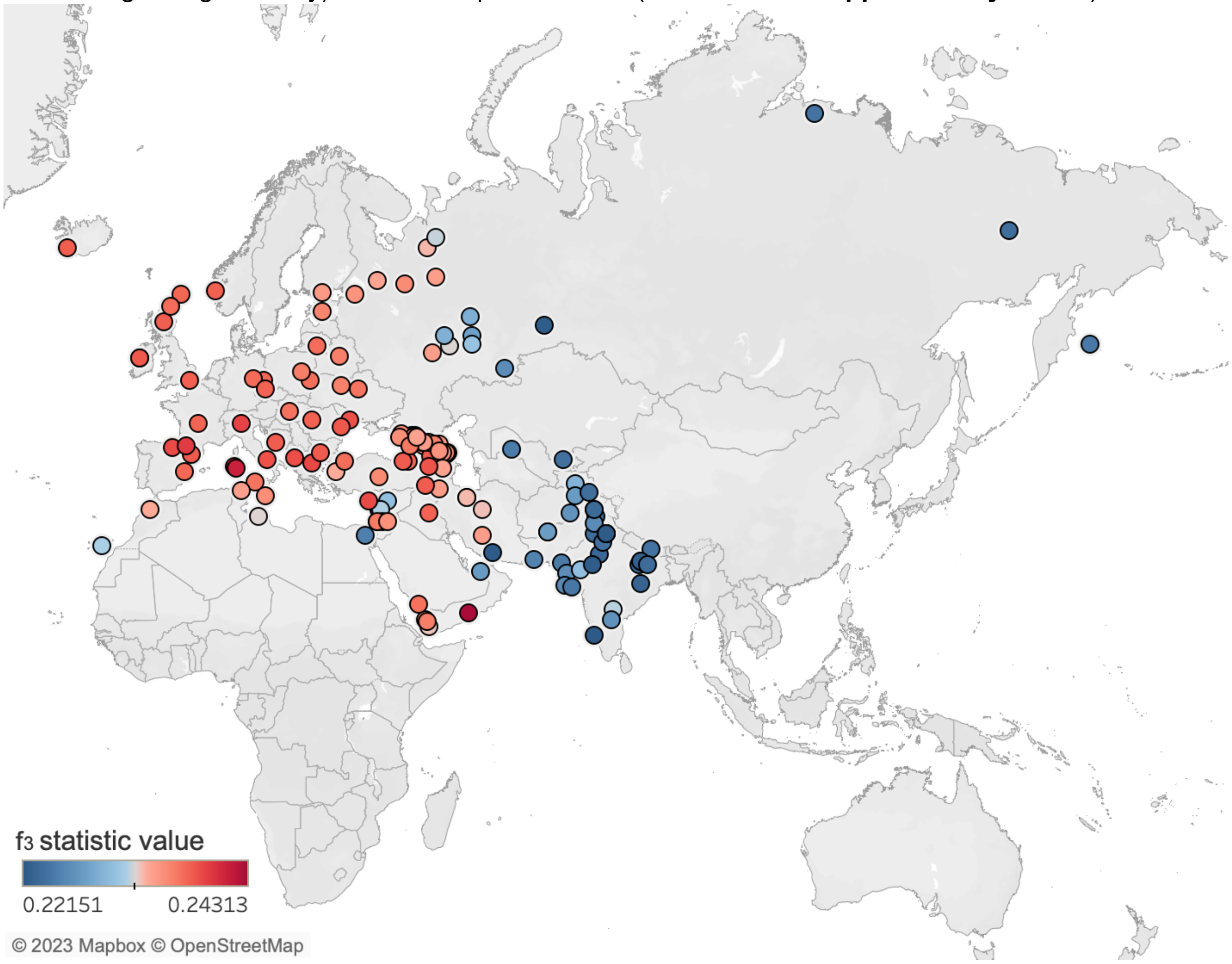

Figure S6. Geographic location of the 150 populations with the highest outgroup f_3-statistics using Mbuti as an outgroup. The color scale is in the bottom left corner, showing the highest amount of allele sharing between Yemen_Soqotri and the Yemeni_Desert group, and highlighting the apparent West Eurasian ancestry of the Soqotri.

Supplementary Note 8: Analysis of Consanguineous Genetic Segments.

We called ROH >4cM for each individual from medieval Soqotra, as well as present-day populations from Yemen, and the Saudi and BedouinB groups (**Figures S7** and **S8**).

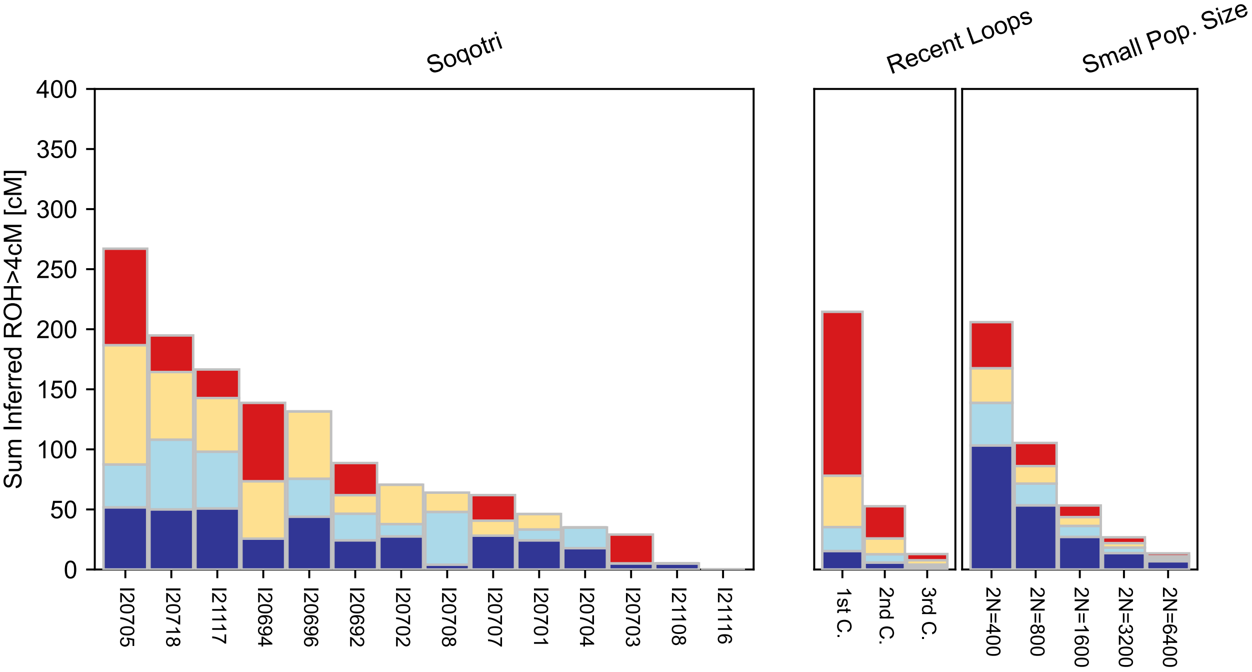

Figure S7. ROH estimates for 14 ancient Soqotri individuals with >300,000 SNPs covered. Each individual is represented by a stacked vertical bar, and the size of each bar is determined by the total ROH >4cM in the genome of this individual. ROH is divided into four length bins: 4–8cM (dark blue), 8–12cM (light blue), 12–20cM (yellow), and >20cM (red). On the right are two plots of expected ROH for offspring of close kin ('C.' denotes cousin) or in small populations (Ringbauer et al., 2021). For all Soqotri ROH data, see **Supplementary Data 10**.

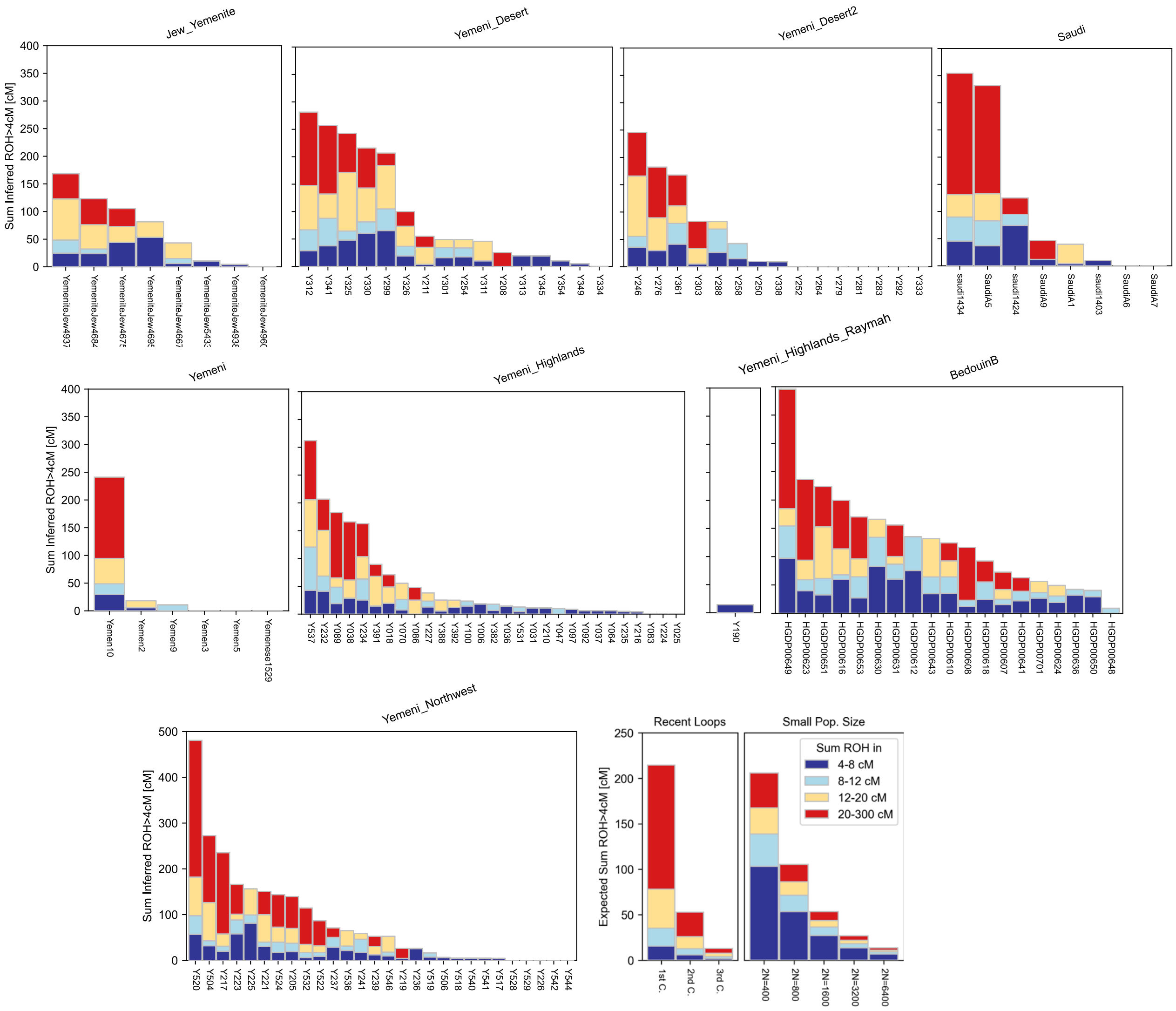

Figure S8. ROH estimates for present-day Arabian and Arabian-related populations. Each individual is represented by a stacked vertical bar, and the size of each bar is determined by the total ROH >4cM in the genome of this individual. ROH is divided into four length bins: 4–8cM, 8–12cM, 12–20cM, and >20cM, color-coded as in the inset legend. On the right in the bottom row are two plots of expected ROH for offspring of close kin ('C.' denotes cousin) or in small populations (Ringbauer et al., 2021).

We then generated individual karyotypes and individual histograms of length distributions with theoretical expectations for these 14 individuals from Soqotra with >300,000 SNPs covered. On the right in **Figure S9** is the karyotype, with positions of ROH segments (maroon marks) on the 22 autosomes (map length is annotated in Morgan). On the left are individual histograms of the ROH lengths of each individual together with expected densities of ROH for select degrees of parental relatedness (Ringbauer et al., 2021).

I20962

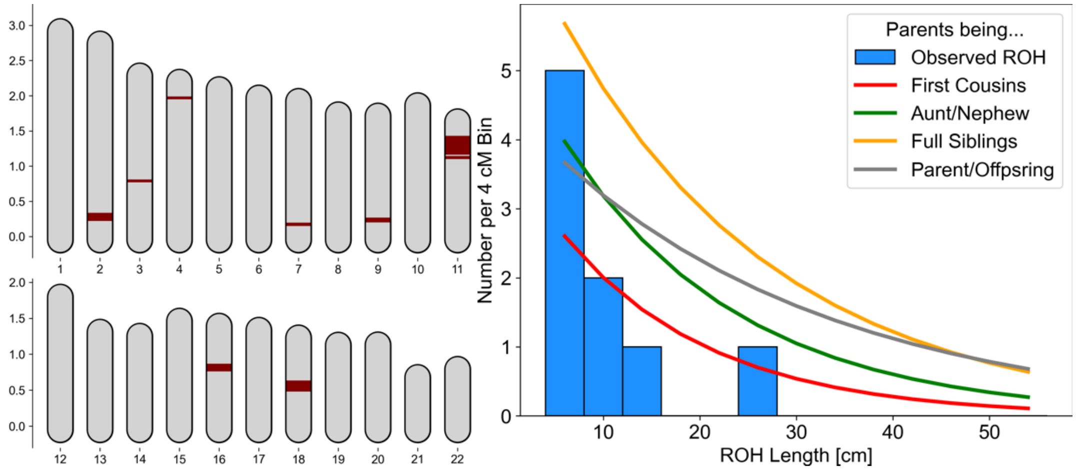

I20694

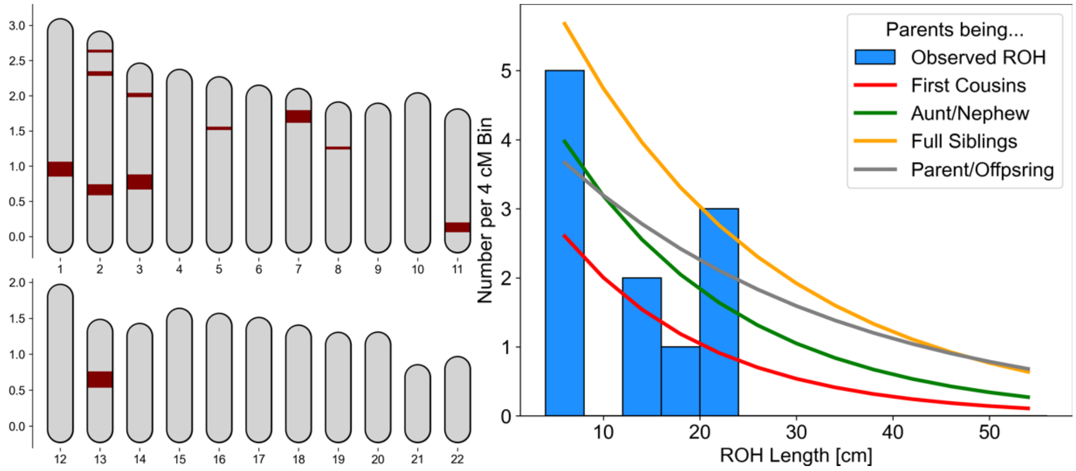

I20696

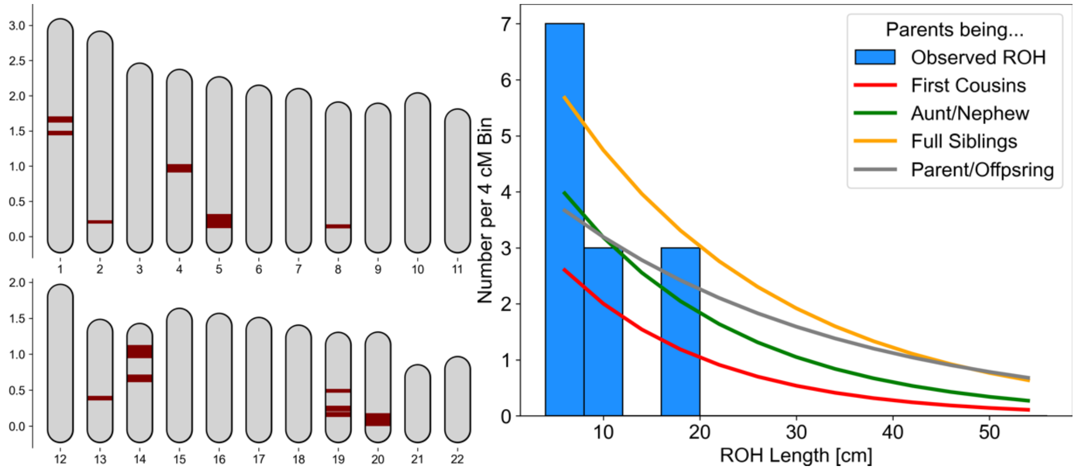

I20701

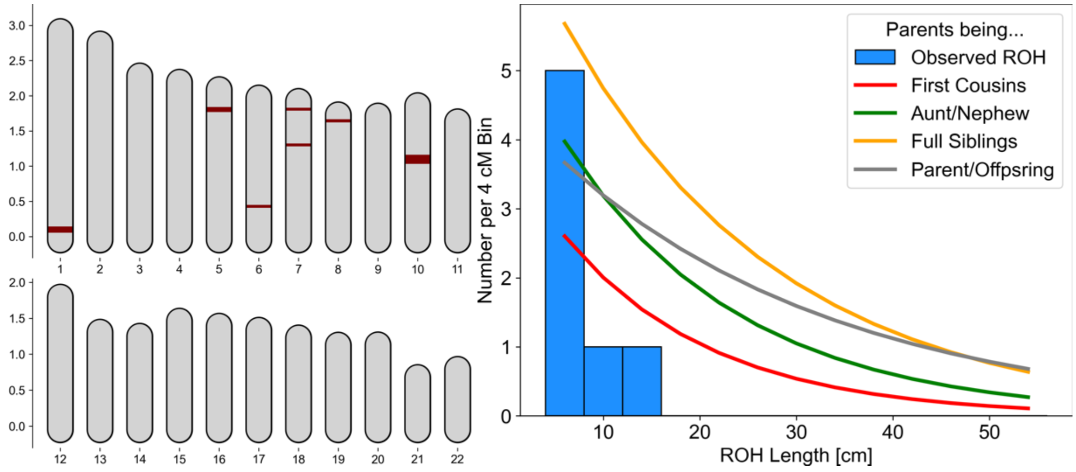

I20702

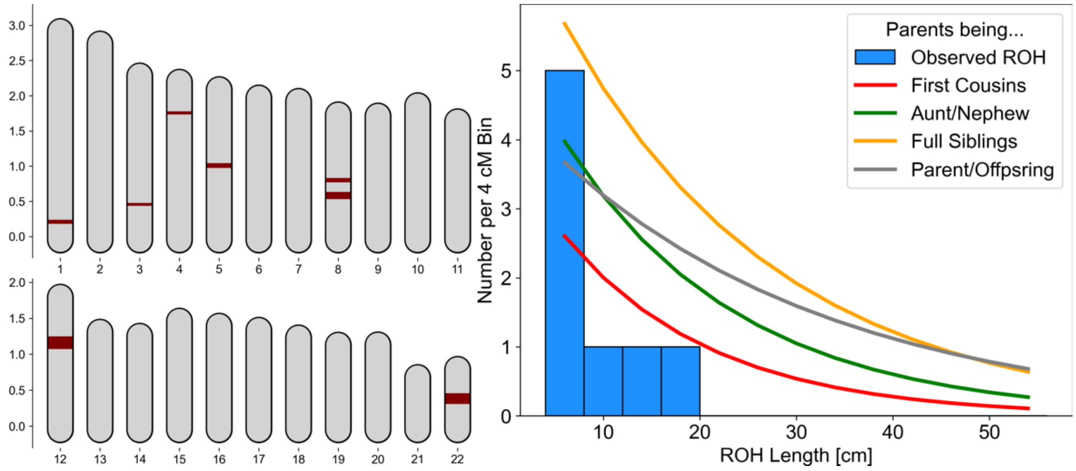

I20703

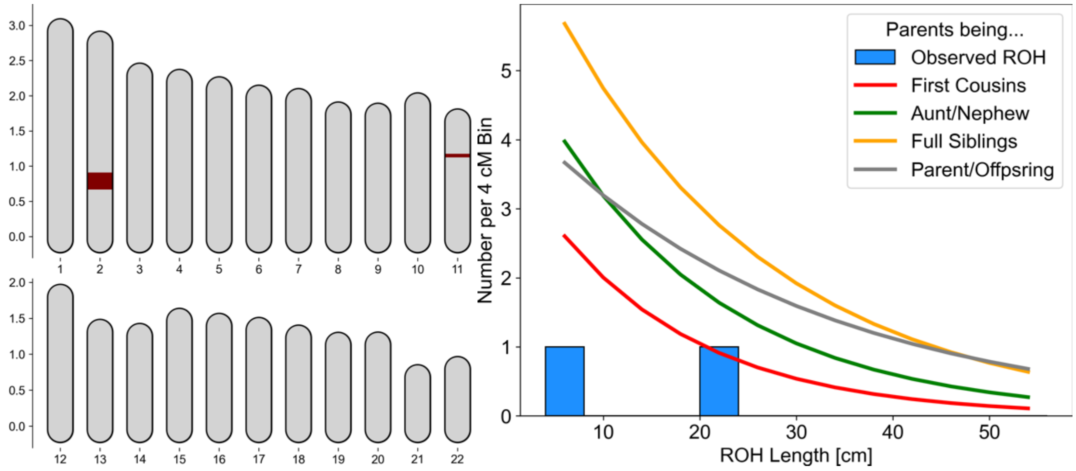

I20704

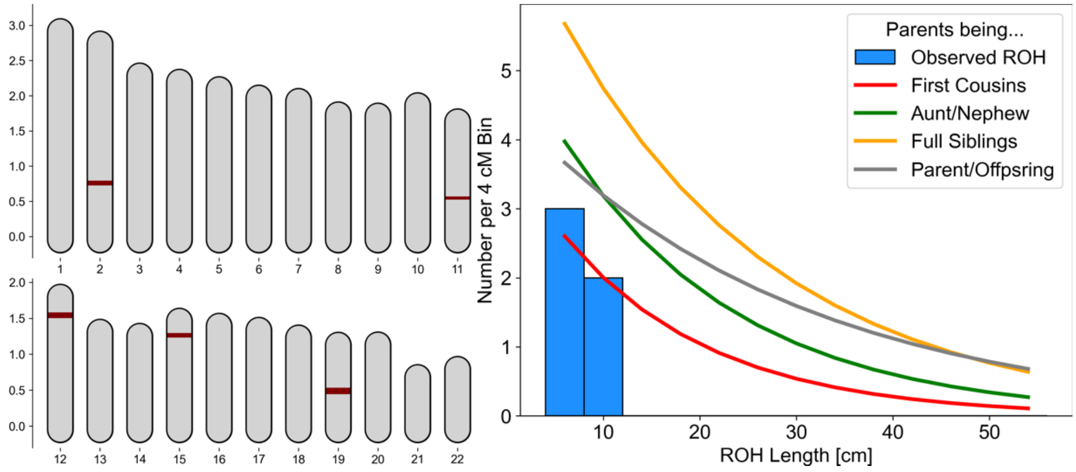

I20705

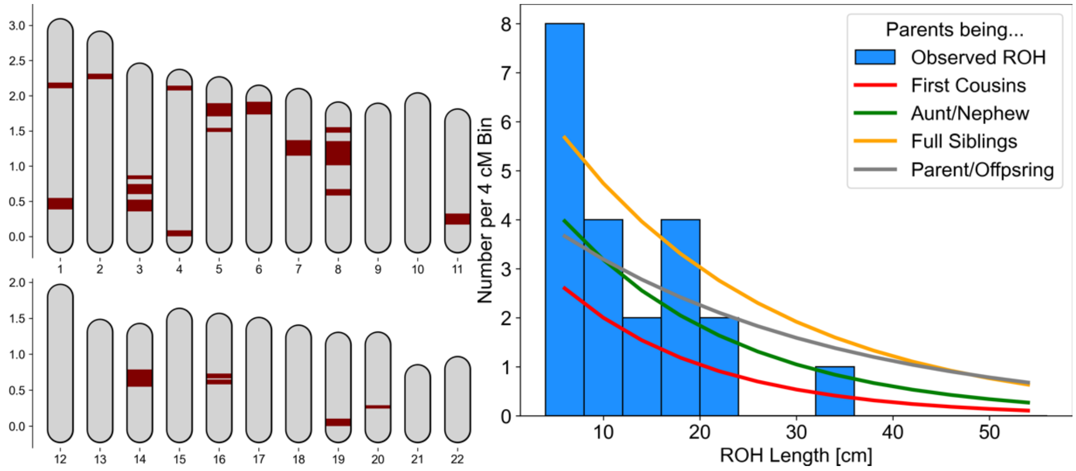

I20707

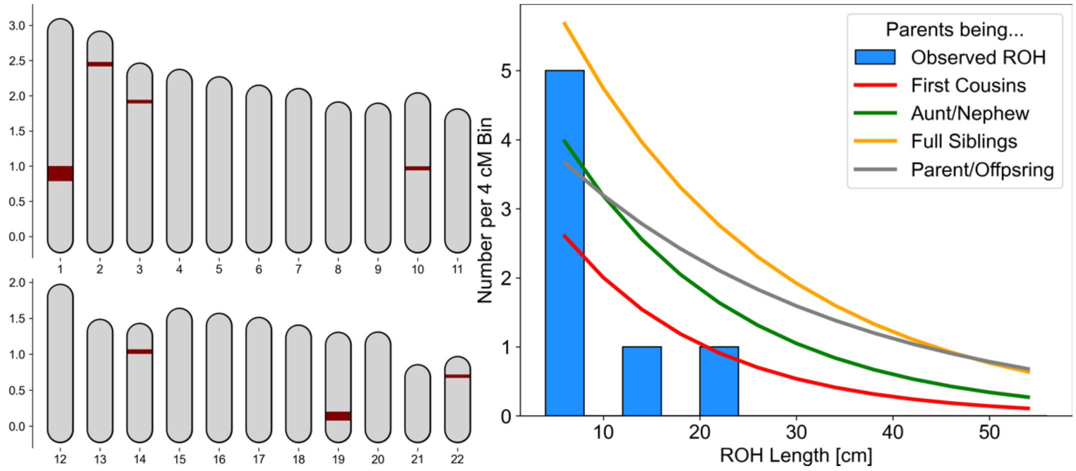

I20708

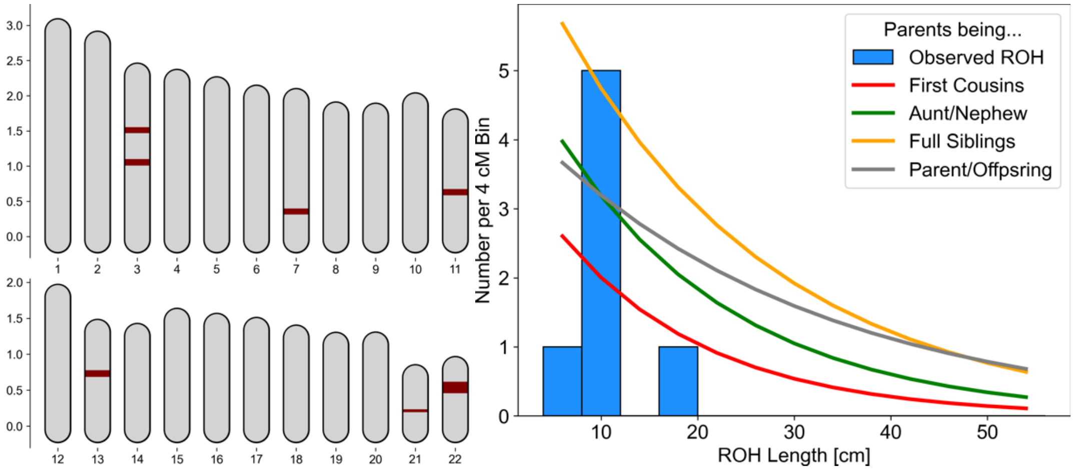

I20718

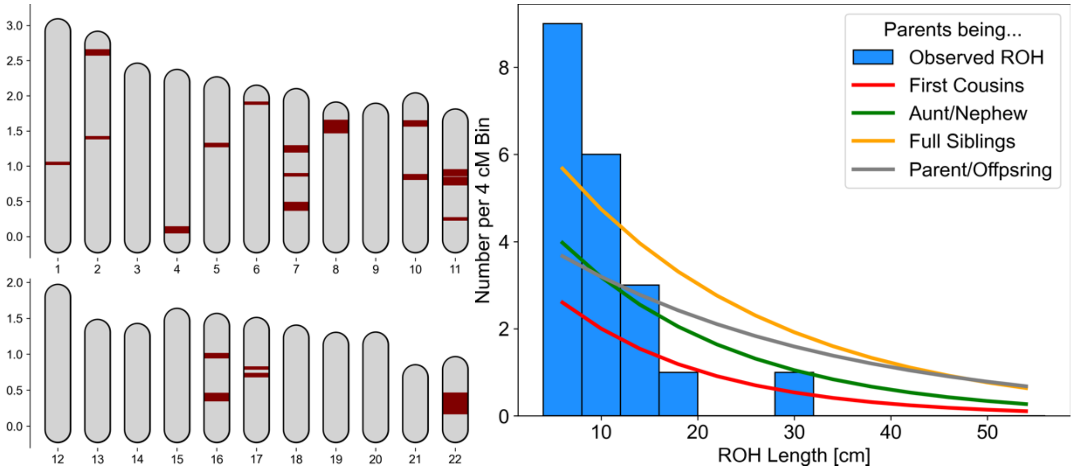

I21108

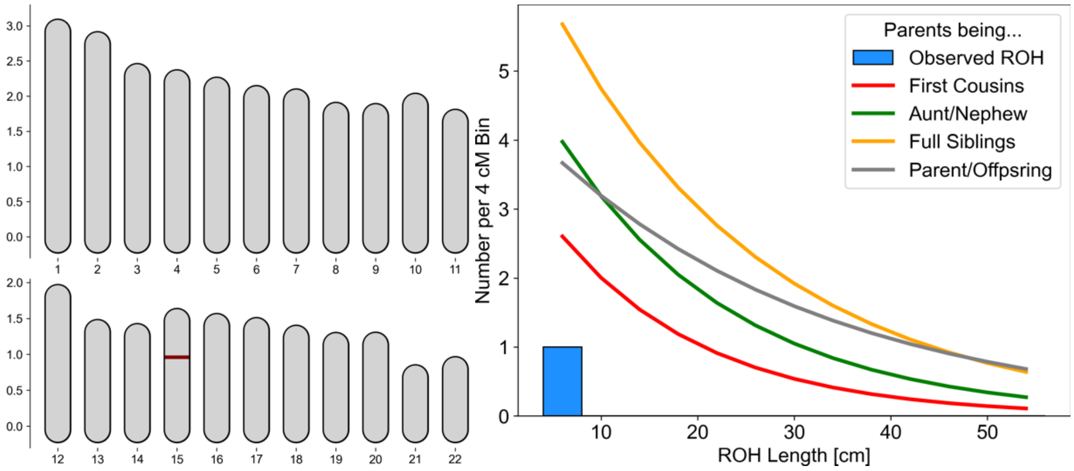

I21116

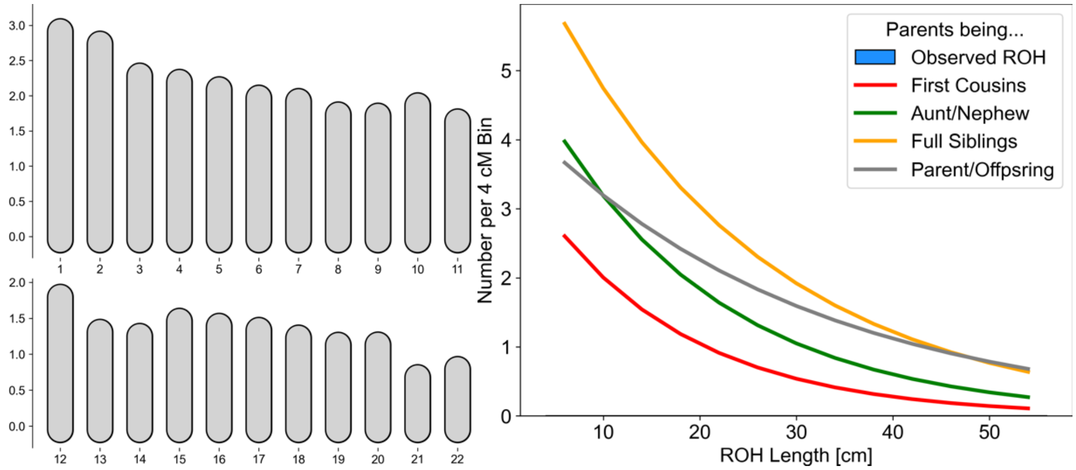

I21117

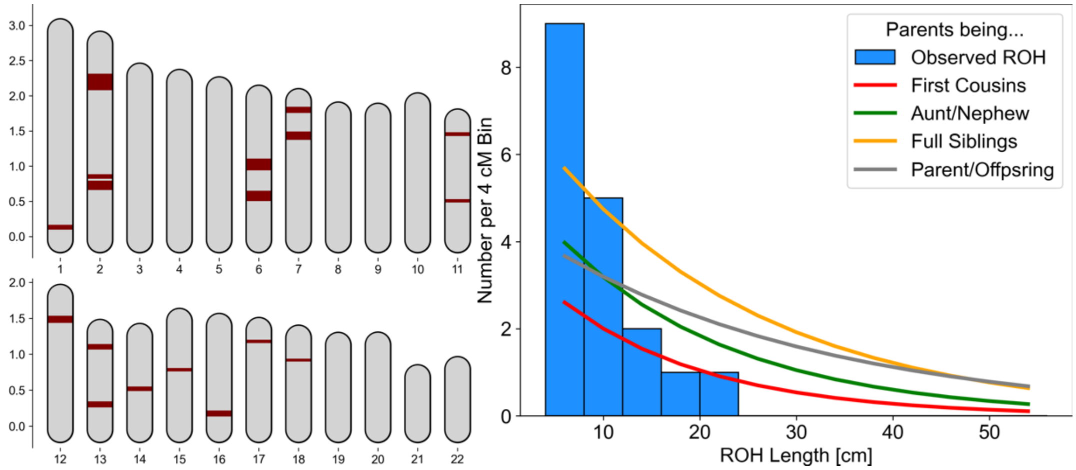

Figure S9. For each of 14 medieval Soqotri individuals with sufficient SNP coverage, we show the individual's karyotype on the right, with positions of ROH segments (maroon marks) on the 22 autosomes (map length is annotated in Morgan) and histograms of the ROH lengths for that individual together with expected densities of ROH for select degrees of parental relatedness, based on analytical calculations (Ringbauer et al., 2021) on the right.

More than half of the medieval Soqotri (8 out of 14) with sufficient coverage (>300,000 SNPs) have at least one long ROH>20cM (maximum of three), reflecting unions within the same extended family; Figure S16 of (Ringbauer et al., 2021) shows that for relationships beyond the 9th-degree (that is, more distant than fourth cousin unions), ROH>20cM is very rare. On the other hand, none of the medieval Soqotri had a total ROH>20cM more than 80.26cM. This is surprising under the hypothesis that Naumkin, 1993's estimate of ~35-40% first cousin unions in present-day Soqotra (**Table S6**) also applied in medieval times, since for first cousin unions, the average sum of ROH segments of >20cM is around 126cM (**Supplementary Data 15**). We would expect a sample of 14 Soqotri individuals to include about 5 products of first cousin unions, and hence one would intuitively expect the maximum ROH observed to be greater than the average seen for first cousin unions.

Table S6. Recreation of Table 7.1 (Forms of Marriage) from (Naumkin, 1993). We note that the Soqotri definition of 'cousin' may have a wide interpretation.

Marriage Partner	% of Respondents	Assessment of relationship in this work
daughter of the father's brother	26.04	cousin
daughter of the father's sister	1.13	cousin
daughter of the mother's brother	6.42	cousin
daughter of the mother's sister	1.89	cousin
father's relative	3.78	cousin
mother's relative	1.13	cousin
distant relative	3.02	unrelated
fellow tribeswoman	26.04	unrelated
inhabitant of same population centre	4.15	unrelated
alien' woman	26.42	unrelated

To test this hypothesis formally, we carried out simulations under the conservative assumption that an individual's parents were either first cousins with probability *F*, or unrelated (this approach over-estimates the first cousin rate when comparing to the real data that includes more distant cousins). For each simulation, we began by using a binomial distribution to identify the number of first cousins in a putative set of 14 individuals, given a probability *F* of first cousin unions. For each first cousin union among the 14, we simulated the sum of ROH>20cM by drawing its value from a distribution of 1,000 simulations of this quantity in first-cousin unions (**Supplementary Data 15**); we assumed ROH>20cM to be 0 for offspring of unrelated people. We ran simulated a set of 14 individuals 100,000 times in this way and counted the proportion where all individuals had summed ROH>20cM less than 80.26cM (the maximum amount of ROH>20cM among the medieval Soqotri identified in the genome of I20705). We then tabulated the proportion of for all F. We find that the observed data (P(max)) are not compatible with the ethnographic value of *F*=0.40 (p-value of 0.007); instead the p=0.05 threshold corresponds to *F*=0.26 (implying a maximum of 26% cousin unions).

F	P(max)	P(mean)
0.00	1.000	1.000
0.01	0.901	0.995
0.02	0.812	0.985
0.03	0.732	0.971
0.04	0.660	0.952
0.05	0.590	0.930
0.06	0.528	0.903
0.07	0.474	0.877
0.08	0.427	0.847
0.09	0.382	0.815
0.10	0.342	0.780
0.11	0.309	0.749
0.12	0.273	0.711
0.13	0.244	0.675
0.14	0.219	0.638
0.15	0.194	0.601
0.16	0.172	0.569
0.17	0.154	0.537
0.18	0.136	0.499
0.19	0.119	0.465
0.20	0.108	0.436
0.21	0.094	0.403
0.22	0.084	0.370
0.23	0.075	0.346
0.24	0.064	0.315
0.25	0.057	0.291
0.26	0.050	0.265
0.27	0.044	0.244
0.28	0.041	0.221
0.29	0.034	0.200
0.30	0.031	0.183
0.31	0.027	0.165
0.32	0.023	0.148
0.33	0.021	0.134
0.34	0.017	0.120
0.35	0.016	0.109
0.36	0.013	0.096
0.37	0.012	0.086
0.38	0.010	0.076
0.39	0.009	0.067
0.40	0.007	0.058
0.41	0.006	0.052
0.42	0.006	0.045
0.43	0.005	0.039
0.44	0.004	0.034
0.45	0.004	0.030
0.46	0.003	0.027
0.47	0.002	0.022
0.48	0.002	0.019
0.49	0.002	0.017
0.50	0.001	0.014

We were concerned that our finding of a lower rate of first cousin unions in the sampled medieval Soqotri than in a sample of modern Soqotri might be an artifact of the simplifying assumption in our simulations that medieval Soqotri were either offspring of first cousins or offspring of unrelated people. This assumption is conservative, however: if we allowed some of the “unrelated” people in simulations to be relatives more distant than first cousins (e.g., second cousins), this would be expected to generate occasional simulated offspring with large $ROH_{>20cM}$, and thus the data would be able to match the simulations by specifying even a lower rate of first cousins. We also considered the possibility that errors in the reconstruction of ROH might have caused us to fail to detect individuals with large amounts of ROH. However, the individuals with the largest amounts of ROH had far more than the minimum required number of 300,000 SNPs used as the threshold for reliable ROH reconstruction based on the extensive validation of the reliability of calling of $ROH_{>20cM}$ segments in the paper that reported the software we used (Ringbauer et al., 2021). A caveat is that the p=0.007 rejection of the modern rate of first cousin unions is dependent on the maximum ROH seen over all samples, a statistic we selected based on being intuitively surprised that none of the medieval Soqotra has $ROH_{>20cM}$ segments in the higher end of the range expected for first cousins (thus there is some degree of multiple hypothesis testing implicit in this finding). We also analyzed more general aspects of the distribution of long ROH segments and found that they were not as effective at ruling out high proportions of first cousin unions as the maximum ROH over all 14 samples (analyses not shown).

Supplementary Note 9: Y Chromosome Haplogroups

Y chromosome haplogroups for all medieval Soqotri males of sufficient coverage are reported in **Supplementary Data 2**.

The only published Y chromosome data from living Soqotri males reports that three-quarters belong to paragroup J-M304 (J* (xJ1,J2)) (Černý et al., 2009); however, the YFull YTree lists no living J* individuals (those negative for downstream SNPs) and instead places the majority of genotyped present-day Soqotri men on the J2b1-Y45076 branch of J2b-M205 (MRCA ~3100 years BP). In line with our Y chromosome calls, J2b1-Y45076 is on the same branch as the haplogroups for the great majority of the medieval Soqotri males we analyze here, with some of the haplogroups to which living Soqotri men belong positioned directly downstream from haplogroups that we document to have been present on Soqotra over 1,000 years ago. For example, YFull reports two present-day Soqotri belonging to J-FT263689, downstream from the J-FT46678 haplogroup called for two men from Zaflah, one of whom lived 675-775 calCE (I20692). Continuity in male-transmitted Y chromosome haplogroups on Soqotra for over a millennium is consistent with a relatively unchanging population throughout time and a system of patrilocality and preference for some level of consanguineous unions.

There is no evidence for recent sub-Saharan African ancestry in the Y chromosome data from medieval Soqotri. While ~10% of males living on Soqotra today belong to Y chromosome haplogroup E (Černý et al., 2009), we find no evidence of it among medieval Soqotri males, who instead all belong to haplogroup J (**Supplementary Data 2**). Although this inference is tentative due to limited sample size, it is consistent with a recent introduction of Y chromosome haplogroup E to the island, possibly as a result of movements that introduced additional ancestry in the modern period. One example could be the Arab slave trade, which increased in intensity throughout the later part of the 1st millennium CE although the African individuals who were forcibly moved during this time may not have left a detectable genetic footprint until the recent move away from slavery on Soqotra only in the mid-20th century when men carrying additional Y lineages may have had more opportunity to contribute to the gene pool.

Supplementary Note 10: Burial Groupings

Here, we describe in greater detail observations made from studying the ancient DNA in burials from 15 *tafone* across six archaeological sites to shed additional light on burial practices in medieval Soqotra. All data can be found in **Supplementary Data 13**.

Observation 1: Closely related individuals were sometimes, but not always, interred in the same *tafone*. These *tafone* also sometimes included unrelated individuals.

- A multi-generational extended family at Zaflah comprising members of both sexes (seven females, 11 males) was interred across eight *tafoni*, along with two unrelated individuals. In one *tafoni* (*Tafone F*), we find a three-generation patriline (I20694 is the father of I20695, who is the father of I20691), as well as a female also fathered by I20694 and a more distant male relative, I20692. I20692 - directly dated using radiocarbon methods as one of the oldest individuals in our dataset (675-775 calCE) is a second- or third-degree relative of I20694 (who is directly dated using radiocarbon methods to 897-1025 calCE) and shares the same Y chromosome haplogroup as the other male members (J2b1), consistent with this individual possibly being the patriarch of this family.
- First-degree relatives I20698 and I20699 (both female and sharing an mtDNA haplogroup) were interred together (*Tafone D*).
- I20706, I20707, and I20708 (two females and one male, respectively), who were all members of the same extended family (Family 1) at Zaflah but were not closely related to one another, were also interred together (*Tafone G*).
- First-degree female relatives I21107 and I20687 were interred together but also with female individual I20688, who did not share a close relationship with either to the limits that we were able to detect but did share the same mtDNA haplogroup (*Tafone J*).
- Second-/third-degree relatives I20704 and I20705 were interred together along with an unrelated female with a different mtDNA haplogroup (I20703) (*Tafone E*).

Observation 2: sometimes unrelated individuals were interred together. We note three cases where three unrelated individuals were interred together in a single *tafone* (in two *tafoni*, there are remains of both sexes while three unrelated males are interred in the third).

- I20702 (a male) and I21108 (his mother) are not buried in the same *tafone*. Instead, the female I21108 was buried with an unrelated female (*Tafone I*), while I20702 was buried a short distance away with an unrelated female (I20700) and another male (I20701) who was not closely related, but was a member of the same extended family (*Tafone C*); I20702 and I20701 belonged to the same Y chromosome haplogroup, carrying the J-Y45447 terminal mutation that is unique to these two individuals relative to other Soqotri males.
- At Sayiher Di Hadiboh, a four-member 'family' was interred across three *tafoni*: a male (I21117) was buried alone (*Tafone M*) while his first-degree female relative (I20724, possibly his mother or sister, based on a shared mtDNA haplogroup) was buried in a separate *tafone* further along the wadi along with another female sharing the same mtDNA haplogroup (I20723, who was a second- or third-degree relative of I21117) (*Tafone N*); another female in this family (I20721) was buried in a separate *tafone* along with a pair of first-degree relatives who formed a separate family (male I21118 and female I20722, plausibly siblings or mother-son based on a shared mtDNA haplogroup) (*Tafone O*).

References

Agranat-Tamir, L., Waldman, S., Martin, M. A. S., Gokhman, D., Mishol, N., Eshel, T., Cheronet, O., Rohland, N., Mallick, S., Adamski, N., Lawson, A. M., Mah, M., Michel, M., Oppenheimer, J., Stewardson, K., Candilio, F., Keating, D., Gamarra, B., Tzur, S., . . . Reich, D. (2020). The Genomic History of the Bronze Age Southern Levant. *Cell*, *181*(5), 1146-1157.

Almarri, M. A., Haber, M., Lootah, R. A., Hallast, P., Al Turki, S., Martin, H. C., Xue, Y., & Tyler-Smith, C. (2021). The genomic history of the Middle East. *Cell*, *184*(18), 4612-4625. e4614.

Biagini, S. A., Solé-Morata, N., Matisoo-Smith, E., Zalloua, P., Comas, D., & Calafell, F. (2019). People from Ibiza: an unexpected isolate in the Western Mediterranean. *European journal of human genetics : EJHG*, *27*(6), 941-951.

Brielle, E. S., Fleisher, J., Wynne-Jones, S., Sirak, K., Broomandkhoshbacht, N., Callan, K., Curtis, E., Iliev, L., Lawson, A. M., Oppenheimer, J., Qiu, L., Stewardson, K., Workman, J. N., Zalzala, F., Ayodo, G., Gidna, A. O., Kabiru, A., Kwekason, A., Mabulla, A. Z. P., . . . Kusimba, C. M. (2023). Entwined African and Asian genetic roots of medieval peoples of the Swahili coast. *Nature*, *615*(7954), 866-873.

Bronk Ramsey, C., & Lee, S. (2013). Recent and planned developments of the program OxCal. *Radiocarbon*, *55*(2), 720-730.

Broushaki, F., Thomas, M. G., Link, V., López, S., van Dorp, L., Kirsanow, K., Hofmanová, Z., Diekmann, Y., Cassidy, L. M., & Díez-del-Molino, D. (2016). Early Neolithic genomes from the eastern Fertile Crescent. *Science*, aaf7943.

Černý, V., Pereira, L., Kujanová, M., Vašíková, A., Hájek, M., Morris, M., & Mulligan, C. J. (2009). Out of Arabia—The settlement of Island Soqotra as revealed by mitochondrial and Y chromosome genetic diversity. *138*(4), 439-447.

Gamba, C., Jones, E. R., Teasdale, M. D., McLaughlin, R. L., Gonzalez-Fortes, G., Mattiangeli, V., Domboroczki, L., Kovari, I., Pap, I., Anders, A., Whittle, A., Dani, J., Raczky, P., Higham, T. F. G., Hofreiter, M., Bradley, D. G., & Pinhasi, R. (2014). Genome flux and stasis in a five millennium transect of European prehistory. *Nature Communications*, *5*(5257).

Hansen, H. B., Damgaard, P. B., Margaryan, A., Stenderup, J., Lynnerup, N., Willerslev, E., & Allentoft, M. E. (2017). Comparing Ancient DNA Preservation in Petrous Bone and Tooth Cementum. *Plos One*, *12*(1), e0170940.

Harney, É., May, H., Shalem, D., Rohland, N., Mallick, S., Lazaridis, I., Sarig, R., Stewardson, K., Nordenfelt, S., Patterson, N., Hershkovitz, I., & Reich, D. (2018). Ancient DNA from Chalcolithic Israel reveals the role of population mixture in cultural transformation. *Nature Communications*, *9*(3336), 1-11.

Jeong, C., Balanovsky, O., Lukianova, E., Kahbatkyzy, N., Flegontov, P., Zaporozhchenko, V., Immel, A., Wang, C.-C., Ixan, O., Khussainova, E., Bekmanov, B., Zaibert, V., Lavryashina, M., Pocheshkhova, E., Yusupov, Y., Agdzhoyan, A., Koshel, S., Bukin, A., Nymadawa, P., . . . Krause, J. (2019). The genetic history of admixture across inner Eurasia. *Nature Ecology & Evolution*, *3*(6), 966-976.

Kivisild, T., Reidla, M., Metspalu, E., Rosa, A., Brehm, A., Pennarun, E., Parik, J., Geberhiwot, T., Usanga, E., & Villems, R. (2004). Ethiopian mitochondrial DNA heritage: tracking gene flow across and around the gate of tears. *The American Journal of Human Genetics*, *75*(5), 752-770.

Lazaridis, I., Alpaslan-Roodenberg, S., Acar, A., Açıkkol, A., Agelarakis, A., Aghikyan, L., Akyüz, U., Andreeva, D., Andrijašević, G., Antonović, D., Armit, I., Atmaca, A., Avetisyan, P., Aytek, A. İ., Bacvarov, K., Badalyan, R., Bakardzhiev, S., Balen, J., Bejko, L., . . . Reich, D. (2022a). The genetic history of the Southern Arc: A bridge between West Asia and Europe. *Science*, *377*(6609), eabm4247.

Lazaridis, I., Alpaslan-Roodenberg, S., Acar, A., Açıkkol, A., Agelarakis, A., Aghikyan, L., Akyüz, U., Andreeva, D., Andrijašević, G., & Antonović, D. (2022b). Ancient DNA from Mesopotamia suggests distinct pre-pottery and pottery Neolithic migrations into Anatolia. *Science*, *377*(6609), 982-987.

Lazaridis, I., Alpaslan-Roodenberg, S., Acar, A., Açıkkol, A., Agelarakis, A., Aghikyan, L., Akyüz, U., Andreeva, D., Andrijašević, G., Antonović, D., Armit, I., Atmaca, A., Avetisyan, P., Aytek, A. İ., Bacvarov, K., Badalyan, R., Bakardzhiev, S., Balen, J., Bejko, L., . . . Reich, D. (2022c). A genetic

probe into the ancient and medieval history of Southern Europe and West Asia. *Science*, *377*(6609), 940-951.
Lazaridis, I., Nadel, D., Rollefson, G., Merrett, D. C., Rohland, N., Mallick, S., Fernandes, D., Novak, M., Gamarra, B., Sirak, K., Connell, S., Stewardson, K., Harney, E., Fu, Q., Gonzalez-Fortes, G., Jones, E. R., Roodenberg, S. A., Lengyel, G., Bocquentin, F., . . . Reich, D. (2016). Genomic insights into the origin of farming in the ancient Near East [Article]. *Nature*, *536*(7617), 419-424.
Lazaridis, I., Patterson, N., Mittnik, A., Renaud, G., Mallick, S., Kirsanow, K., Sudmant, P. H., Schraiber, J. G., Castellano, S., & Lipson, M. (2014). Ancient human genomes suggest three ancestral populations for present-day Europeans. *Nature*, *513*(7518), 409-413.
López, S., Tarekegn, A., Band, G., van Dorp, L., Bird, N., Morris, S., Oljira, T., Mekonnen, E., Bekele, E., Blench, R., Thomas, M. G., Bradman, N., & Hellenthal, G. (2021). Evidence of the interplay of genetics and culture in Ethiopia. *Nature Communications*, *12*(1), 3581.
Mathieson, I., Lazaridis, I., Rohland, N., Mallick, S., Patterson, N., Roodenberg, S. A., Harney, E., Stewardson, K., Fernandes, D., Novak, M., Sirak, K., Gamba, C., Jones, E. R., Llamas, B., Dryomov, S., Pickrell, J., Arsuaga, J. L., de Castro, J. M. B., Carbonell, E., . . . Reich, D. (2015). Genome-wide patterns of selection in 230 ancient Eurasians [Article]. *Nature*, *528*(7583), 499-503.
Nakatsuka, N., Moorjani, P., Rai, N., Sarkar, B., Tandon, A., Patterson, N., Bhavani, G. S., Girisha, K. M., Mustak, M. S., Srinivasan, S., Kaushik, A., Vahab, S. A., Jagadeesh, S. M., Satyamoorthy, K., Singh, L., Reich, D., & Thangaraj, K. (2017). The promise of discovering population-specific disease-associated genes in South Asia. *Nature genetics*, *49*(9), 1403-1407.
Narasimhan, V. M., Patterson, N., Moorjani, P., Rohland, N., Bernardos, R., Mallick, S., Lazaridis, I., Nakatsuka, N., Olalde, I., Lipson, M., Kim, A. M., Olivieri, L. M., Coppa, A., Vidale, M., Mallory, J., Moiseyev, V., Kitov, E., Monge, J., Adamski, N., . . . Reich, D. (2019). The formation of human populations in South and Central Asia. *Science*, *365*(6457), eaat7487.
Naumkin, V. V. a. (1993). *Island of the Phoenix: An ethnographic study of the people of Socotra*. Paul & Company Pub Consortium.
Patterson, N., Moorjani, P., Luo, Y., Mallick, S., Rohland, N., Zhan, Y., Genschoreck, T., Webster, T., & Reich, D. (2012). Ancient admixture in human history. *Genetics*, *192*(3), 1065-1093.
Pinhasi, R., Fernandes, D., Sirak, K., Novak, M., Connell, S., Alpaslan-Roodenberg, S., Gerritsen, F., Moiseyev, V., Gromov, A., Raczky, P., Anders, A., Pietrusewsky, M., Rollefson, G., Jovanovic, M., Trinhhoang, H., Bar-Oz, G., Oxenham, M., Matsumura, H., & Hofreiter, M. (2015). Optimal Ancient DNA Yields from the Inner Ear Part of the Human Petrous Bone. *Plos One*, *10*(6), e0129102.
Prendergast, M. E., Lipson, M., Sawchuk, E. A., Olalde, I., Ogola, C. A., Rohland, N., Sirak, K. A., Adamski, N., Bernardos, R., Broomandkhoshbacht, N., Callan, K., Culleton, B. J., Eccles, L., Harper, T. K., Lawson, A. M., Mah, M., Oppenheimer, J., Stewardson, K., Zalzala, F., . . . Reich, D. (2019). Ancient DNA reveals a multistep spread of the first herders into sub-Saharan Africa. *Science*, *365*(6448), eaaw6275. https://doi.org/10.1126/science.aaw6275
Reich, D., Thangaraj, K., Patterson, N., Price, A. L., & Singh, L. (2009). Reconstructing Indian population history. *Nature*, *461*(7263), 489-494.
Reimer, P. J., Austin, W. E., Bard, E., Bayliss, A., Blackwell, P. G., Ramsey, C. B., Butzin, M., Cheng, H., Edwards, R. L., & Friedrich, M. (2020). The IntCal20 Northern Hemisphere radiocarbon age calibration curve (0–55 cal kBP). *Radiocarbon*, *62*(4), 725-757.
Ringbauer, H., Novembre, J., & Steinrücken, M. (2021). Parental relatedness through time revealed by runs of homozygosity in ancient DNA. *Nature Communications*, *12*(1), 5425. https://doi.org/10.1038/s41467-021-25289-w
Schuenemann, V. J., Peltzer, A., Welte, B., van Pelt, W. P., Molak, M., Wang, C.-C., Furtwängler, A., Urban, C., Reiter, E., Nieselt, K., Teßmann, B., Francken, M., Harvati, K., Haak, W., Schiffels, S., & Krause, J. (2017). Ancient Egyptian mummy genomes suggest an increase of Sub-Saharan African ancestry in post-Roman periods [Article]. *Nature Communications*, *8*(1), 1-11.
Skoglund, P., Posth, C., Sirak, K., Spriggs, M., Valentin, F., Bedford, S., Clark, G. R., Reepmeyer, C., Petchey, F., & Fernandes, D. (2016). Genomic insights into the peopling of the Southwest Pacific. *Nature*.

Vyas, D. N., Al-Meeri, A., & Mulligan, C. J. (2017). Testing support for the northern and southern dispersal routes out of Africa: an analysis of Levantine and southern Arabian populations. *American Journal of Physical Anthropology*, *164*(4), 736-749.
